# Exact Formula of the Total Quasi-Steady State Approximation in Competitive Target-Mediated Drug Disposition

**DOI:** 10.34133/csbj.0223

**Published:** 2026-10-08

**Authors:** Taehong Kim, Jaeyun Cha, Hyeseon Jeon, Hwi-yeol Yun, Dongju Lim, Jae Kyoung Kim

**Affiliations:** ^1^ School of Freshman, KAIST, Daejeon 34141, Republic of Korea.; ^2^ College of Pharmacy, Chungnam National University, Daejeon, Republic of Korea.; ^3^ Bio-AI Convergence, Chungnam National University, Daejeon, Republic of Korea.; ^4^ Department of Mathematical Sciences, KAIST, Daejeon 34141, Republic of Korea.; ^5^ Biomedical Mathematics Group, Pioneer Research Center for Mathematical and Computational Sciences, Institute for Basic Science, Daejeon 34126, Republic of Korea.; ^6^ Department of Medicine, College of Medicine, Korea University, Seoul 02841, Republic of Korea.

## Abstract

Competitive target-mediated drug disposition (competitive TMDD) arises when 2 drugs compete for the same target receptor. The full competitive TMDD model characterizes these dynamics; yet, its complexity motivated the use of a reduced model, which is invalid under high receptor concentrations. While this problem can be resolved by using the total quasi-steady state approximation, which remains valid for broad parameter regimes, the exact formula of the total quasi-steady state approximation-based reduced model—competitive qTMDD—remained unknown for 15 years, as it requires solving a cubic equation and identifying a biologically meaningful solution. Consequently, researchers have relied on computationally expensive numerical approximations, limiting practical applicability. Here, we derive—for the first time—a real-valued exact formula for competitive qTMDD by leveraging analytic properties of cubic equations and geometric characteristics of their roots in the complex plane. This formulation improved computational speed by approximately 18-fold compared to numerical approximation methods, enabling competitive qTMDD-based Bayesian inference that was previously impractical. Applied to clinical trial data for anakinra and rhIL-7-hyFc, competitive qTMDD estimated pharmacological parameters comparable to those from the full competitive TMDD model while requiring only approximately 28% of the computation time. Importantly, it maintains robust accuracy even at low sampling density, whereas previous reduced models exhibit bias. By ensuring precise biological interpretation of drug systems even in complex real-world scenarios, the exact formula of competitive qTMDD has the potential to streamline the drug development and clinical testing process. Our exact formula also consists entirely of real-valued terms, allowing seamless integration into existing pharmacometrics software.

## Introduction

Competitive target-mediated drug disposition (competitive TMDD) describes a pharmacokinetic system in which 2 ligands compete for a shared pharmacologic target [1–5]. Such competition may arise between an exogenous therapeutic drug and an endogenous ligand or between 2 therapeutic compounds that bind to the same target. The pharmacokinetic importance of this competition is context-dependent and is determined by factors including the relative ligand concentrations, their binding affinities, and the abundance of the shared target. Thus, competition may be negligible in some regimes but substantially alter drug disposition in others. For example, 2 soluble epoxide hydrolase inhibitors have been shown to compete for the same pharmacological target, with displacement of one inhibitor by the other producing measurable changes in its concentration–time profile [6]. These observations motivate quantitative characterization of competitive TMDD and, in particular, analytical approaches that can describe how competition modifies the disposition of interacting ligands.

The competitive TMDD model is represented by a system of ordinary differential equations (ODEs) that describes interactions between 2 competing drugs, a receptor, and their complexes. This system has been analyzed using various model reduction approaches including computational singular perturbation, geometric singular perturbation theory, blow-up methods, and matched asymptotic analysis [7–10]. One of the renowned approaches is the quasi-steady state approximation (QSSA), which reduces the number of variables, thereby increasing computational efficiency and enabling parameter estimation with limited data [11]. Previously, standard QSSA (sQSSA)—which assumes that the drug–receptor complex concentration changes rapidly compared to the drug concentration and thus reaches the quasi-steady state (QSS)—was widely used [12–16]; however, it fails to provide an accurate approximation when the total receptor concentration (free receptor concentration plus the 2 drug–receptor complex concentrations) is not sufficiently small relative to the drug concentration and Michaelis constant scales [16–19]—a situation frequently encountered in cases such as the interaction between omalizumab and immunoglobulin E [20]. To address this limitation, the total QSSA (tQSSA)—which assumes that the drug–receptor complex concentration changes rapidly compared to the total drug concentration (drug concentration plus the drug–receptor complex concentration)—has been proposed as an alternative that achieves greater accuracy across a broader range of conditions [11,21–26].

Although tQSSA shows high approximation accuracy in broader parameter regimes relative to sQSSA, its implementation in the competitive TMDD system is challenging, as it requires solving a cubic equation and selecting the biologically meaningful solution (i.e., the solution where all variables have non-negative concentrations) among the 3 possible roots [1,24,27]. Although the smallest root is proven to be biologically meaningful, identifying the smallest root remains nontrivial [1]. Consequently, the tQSSA formulation of competitive TMDD—competitive qTMDD—has relied on numerical approximation methods since its initial development nearly 15 years ago [1–3]. However, these methods can impose substantial computational costs to maintain accuracy, limiting their practical use in real-world applications. This underscores the urgent need for deriving an exact formula of competitive qTMDD to achieve both accuracy and efficiency in real-world competitive qTMDD applications.

In this study, we derived an exact analytical formula for competitive qTMDD by exploiting the geometric properties of the cubic roots in the complex plane, thereby providing the explicit solution that had remained unavailable since the model was first proposed. The resulting competitive qTMDD model closely approximated the full competitive TMDD system established in previous studies, while achieving computation speeds approximately 18-fold faster than previous numerical methods. As a result, parameter estimation, which previously took over 5.5 h, could be completed in just 15 min of computation. Furthermore, when we applied competitive qTMDD to real-world anakinra and rhIL-7-hyFc data, we accurately estimated the pharmacological parameters. In particular, the performance of competitive qTMDD remained consistently accurate regardless of data sparsity, in contrast to the substantial inaccuracy observed in sQSSA-based simplification under conditions of limited early-phase sampling. By providing a highly efficient and accurate means of parameter estimation for competitive drug systems, the exact formula of competitive qTMDD has the potential to enhance the accuracy of drug dosing and biological interpretation of drug systems, consequently reducing the time and costs associated with drug development and testing.

## Materials and Methods

### Derivation of competitive mTMDD and qTMDD equations

Competitive TMDD is represented by the system of ODEs following [1,11]:

$$\frac{d}{\mathrm{dt}}C_{i}=-k_{\mathrm{el},i}C_{i}-k_{\mathrm{on},i}C_{i}\cdot \left(R_{\mathrm{tot}}-RC_{1}-RC_{2}\right)+k_{\mathrm{off},i}RC_{i},$$ (1)

$$\frac{d}{\mathrm{dt}}RC_{i}=k_{\mathrm{on},i}C_{i}\left(R_{\mathrm{tot}}-RC_{1}-RC_{2}\right)-\left(k_{\mathrm{off},i}+k_{\text{int},i}\right)RC_{i},$$ (2)

for i=1,2, where $C_{i}$ is the drug concentration, $RC_{i}$ is the drug–receptor complex concentration, $R_{\mathrm{tot}}$ is the total receptor concentration, $k_{\mathrm{el},i}$ is the drug elimination constant, $k_{\mathrm{on},i}$ is the association rate constant, $k_{\mathrm{off},i}$ is the dissociation rate constant, and $k_{\text{int},i}$ is the drug–receptor complex internalization constant. Following the previous study [11], all variables and parameters were expressed in arbitrary units, the total receptor concentration was kept constant, and the initial conditions are given as $C_{i}\left(0\right)=C_{i,0}$, $RC_{i}\left(0\right)=0$ for i=1,2.

To derive the standard QSSA of competitive TMDD (competitive mTMDD; Fig. 1), we assume that the drug–receptor complex concentration $RC_{i}$ changes rapidly compared to the drug concentration $C_{i}$ and thus reaches the QSS where $\mathrm{dR}C_{i}/\mathrm{dt}=0$.

**Fig. 1.**
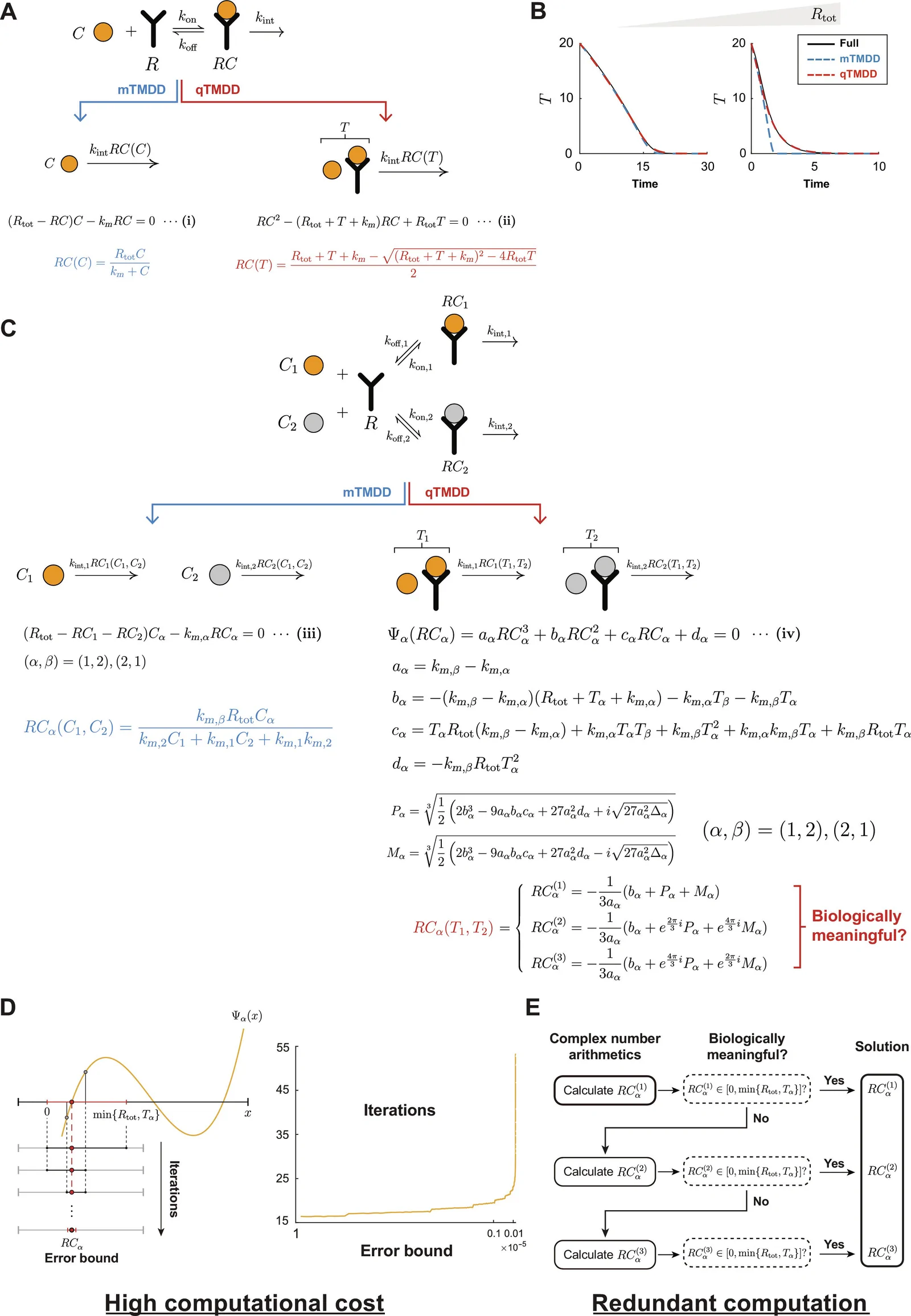
The exact formula of competitive qTMDD is largely unknown, leading to the use of inefficient alternative methods. (A) Single-drug target-mediated drug disposition (TMDD) describes the binding of the drug (C) with the receptor (R), forming the drug–receptor complex (RC). This system can be simplified into 2 models: mTMDD and qTMDD. mTMDD only requires solving a simple linear equation (i), whereas qTMDD involves solving a quadratic equation (ii). This quadratic equation leads to 2 candidate solutions ($RC^{\left(1\right)}$ and $RC^{\left(2\right)}$), among which the unique biologically meaningful solution (i.e., the positive solution smaller than the total drug concentration [T=C+RC] and total receptor concentration $\left[R_{\mathrm{tot}}=R+\mathrm{RC}\right]$) can be easily determined. (B) The plot showing the trajectory of total drug concentration (T=C+RC) simulated from the full TMDD (black line), mTMDD (blue dashed line), and qTMDD (red dashed line) under the low total receptor concentration (left) and high total receptor concentration (right). While the simpler mTMDD is accurate under the low receptor concentration, it becomes inaccurate at high total receptor concentrations. In contrast, the qTMDD remains accurate under both conditions. (C) Competitive TMDD describes the binding of 2 drugs ($C_{1},C_{2}$) with the receptor (R), competing with each other to form the drug–receptor complexes ($RC_{1},RC_{2}$). This system can also be simplified into mTMDD and qTMDD models. mTMDD only requires solving a linear system of equations (iii), whereas the qTMDD involves solving 2 cubic equations (iv). Each of the cubic equations leads to 3 candidate solutions ($RC^{\left(1\right)},RC^{\left(2\right)},$ and $RC^{\left(3\right)}$), but unlike the quadratic equation case (ii), distinguishing the unique biologically meaningful solution is mathematically unresolved, making an explicit form of competitive qTMDD remain unknown. (D) Consequently, the solution for the competitive qTMDD has been calculated relying on alternative methods. One representative example is the bisection method, which iteratively approximates the solution until reaching a desired error bound (left). However, as the error bound becomes stricter, the computational time increases exponentially (right). (E) Another method, the comparison method, calculates each of 3 candidates and identifies the biologically meaningful one. However, this method requires complex number computations, which can be challenging to implement in pharmacokinetic software such as nonlinear mixed effects modeling (NONMEM). In addition, the comparison method involves redundant calculations, increasing computational time.

Then, we obtain the following system of linear equations in $RC_{1}$ and $RC_{2}$:

$$C_{1}\left(R_{\mathrm{tot}}-RC_{1}-RC_{2}\right)-k_{m,1}RC_{1}=0,$$ (3)

$$C_{2}\left(R_{\mathrm{tot}}-RC_{1}-RC_{2}\right)-k_{m,2}RC_{2}=0,$$ (4)

where $k_{m,i}=\left(k_{\mathrm{off},i}+k_{\text{int},i}\right)/k_{\mathrm{on},i}$ for each i=1,2. This system of linear equations can be solved easily as the following:

$$RC_{1}\left(C_{1},\;C_{2}\right)=\frac{k_{m,2}R_{\mathrm{tot}}C_{1}}{k_{m,2}C_{1}+k_{m,1}C_{2}+k_{m,1}k_{m,2}},$$ (5)

$$RC_{2}\left(C_{1},\;C_{2}\right)=\frac{k_{m,1}R_{\mathrm{tot}}C_{2}}{k_{m,2}C_{1}+k_{m,1}C_{2}+k_{m,1}k_{m,2}}.$$ (6)

To derive the tQSSA of competitive TMDD (competitive qTMDD; Fig. 1), we assume that the drug–receptor complex concentration $RC_{i}$ changes rapidly compared to the total drug concentration $T_{i}=C_{i}+RC_{i}$ and thus reaches the QSS where $\mathrm{dR}C_{i}/\mathrm{dt}=0$ as done in the previous studies [1,11]. By substituting $C_{i}$ with $T_{i}-RC_{i}$ in the equation $\mathrm{dR}C_{i}/\mathrm{dt}=0$, we obtain the following system of quadratic equations in $RC_{1}$ and $RC_{2}$:

$$\left(T_{1}-RC_{1}\right)\left(R_{\mathrm{tot}}-RC_{1}-RC_{2}\right)-k_{m,1}RC_{1}=0,$$ (7)

$$\left(T_{2}-RC_{2}\right)\left(R_{\mathrm{tot}}-RC_{1}-RC_{2}\right)-k_{m,2}RC_{2}=0,$$ (8)

where $k_{m,i}=\left(k_{\mathrm{off},i}+k_{\text{int},i}\right)/k_{\mathrm{on},i}$ for each i=1,2. By simplifying Eqs. (7) and (8), $RC_{1}$ and $RC_{2}$ can be expressed in terms of each other as follows ($RC_{1}–RC_{2}$ relation):

$$RC_{1}=R_{\mathrm{tot}}-RC_{2}-\frac{k_{m,2}RC_{2}}{T_{2}-RC_{2}}=\rho_{2}\left(RC_{2}\right)$$ (9)

$$RC_{2}=R_{\mathrm{tot}}-RC_{1}-\frac{k_{m,1}RC_{1}}{T_{1}-RC_{1}}=\rho_{1}\left(RC_{1}\right).$$ (10)

By substituting Eqs. (9) and (10) into Eqs. (7) and (8), we obtain 2 cubic equations where:

$$\Psi_{1}\left(RC_{1}\right)=0,\Psi_{2}\left(RC_{2}\right)=0$$

$$\Psi_{1}\left(x\right)=a_{1}x^{3}+b_{1}x^{2}+c_{1}x+d_{1}$$ (11)

$$a_{1}=k_{m,2}-k_{m,1}$$ (12)

$$b_{1}=-\left(k_{m,2}-k_{m,1}\right)\left(R_{\mathrm{tot}}+T_{1}+k_{m,1}\right)-k_{m,1}T_{2}-k_{m,2}T_{1}$$ (13)

$$c_{1}=T_{1}R_{\mathrm{tot}}\left(k_{m,2}-k_{m,1}\right)+k_{m,1}T_{1}T_{2}+k_{m,2}T_{1}^{2}+k_{m,1}k_{m,2}T_{1}+k_{m,2}R_{\mathrm{tot}}T_{1}$$ (14)

$$d_{1}=-k_{m,2}R_{\mathrm{tot}}T_{1}^{2}$$ (15)

and $\Psi_{2}\left(x\right)$ is obtained by interchanging 1 and 2 in the indices of $\Psi_{1}\left(x\right)$.

Solving the resulting cubic equation for each drug–receptor complex and selecting the biologically meaningful solution yields the exact formula of competitive qTMDD, which will be used in the following ODE:

$$\frac{d}{\mathrm{dt}}T_{i}=-k_{\mathrm{el},i}\left(T_{i}-RC_{i}\left(T_{1},\;T_{2}\right)\right)-k_{\text{int},i}RC_{i}\left(T_{1},\;T_{2}\right)$$ (16)

for i=1,2.

### Implementation of the bisection method

The bisection method computes the biologically meaningful root of the competitive qTMDD by approximating the root of $\Psi_{1}\left(x\right)$ in the biologically meaningful region ($\left[0,\;\min\left\{R_{\mathrm{tot}},\;T_{1}\right\}\right]$) by reducing the interval through several iterations (Fig. 1D). The pseudo-code for the method can be found in Algorithm 1 in Text S1.

Here, the error bound was set to $\varepsilon =10^{-16}$ and the maximum iteration n was set to 54, following a previous study [1]. The MATLAB code for the bisection method can be found in File S1.

### Implementation of the comparison method

The comparison method computes all 3 roots of $\Psi_{1}\left(x\right)$ and finds the biologically meaningful solution by checking whether each solution is in the biologically meaningful region (Fig. 1E) $\left[0,\;\min\left\{R_{\mathrm{tot}},T_{1}\right\}\right]$. While 3 roots of $\Psi_{1}\left(x\right)$ can be computed directly using the cubic formula, this method requires the use of software that supports complex number arithmetic since the cubic formula contains complex-valued terms. Therefore, we implemented the comparison method using the “roots” function in MATLAB as done in [1]. The MATLAB code for the comparison method can be found in File S1.

### Derivation of a biologically meaningful root for competitive qTMDD

To find a biologically meaningful solution of $\Psi_{1}\left(RC_{1}\right)=0\;\text{and}\;\Psi_{2}\left(RC_{2}\right)=0$ (Fig. 2), which is the solution satisfying $\left(RC_{1},\;RC_{2}\right)\in \left[0,\;\text{min}\left\{R_{\mathrm{tot}},\;T_{1}\right\}\right]\times \left[0,\;\text{min}\left\{R_{\mathrm{tot}},\;T_{2}\right\}\right]$ , we considered 3 different cases: $k_{m,1}=k_{m,2}$, $k_{m,1}<k_{m,2}$, and $k_{m,2}<k_{m,1}$.

**Fig. 2.**
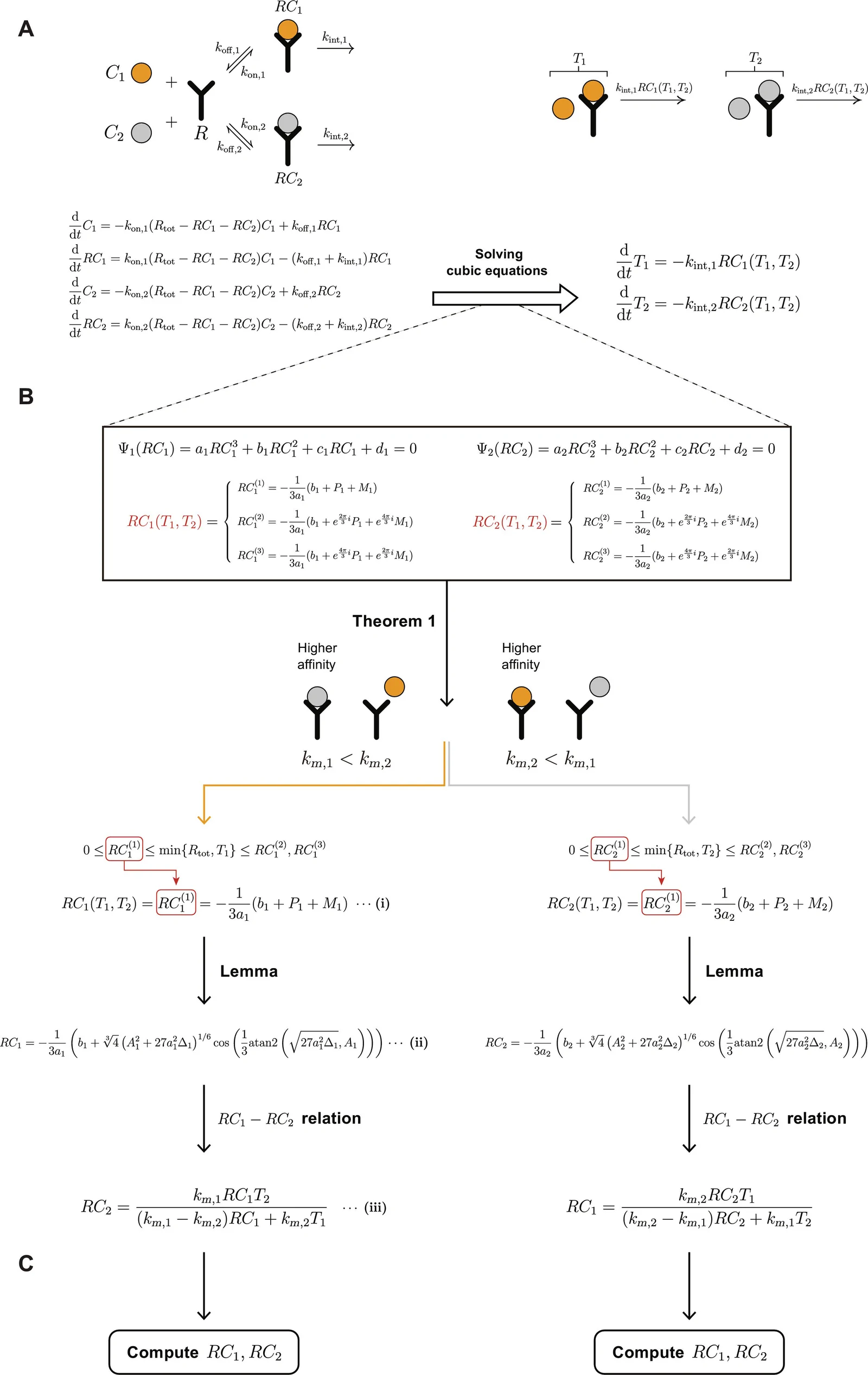
The biologically meaningful solutions for competitive qTMDD can be explicitly derived. (A) To simplify the full competitive target-mediated drug disposition (TMDD) model to the competitive qTMDD model, we provide a mathematical analysis for solving cubic equations and identify biologically meaningful solutions from the candidate solutions. (B) Deriving the competitive qTMDD model requires solving 2 symmetric cubic equations $\Psi_{1}\left(RC_{1}\right)=0$ and $\Psi_{2}\left(RC_{2}\right)=0,$ which yield 3 candidate solutions each for $RC_{1}$ and $RC_{2}$ (shown in the box). To identify the biologically meaningful solutions, we derive inequalities among 3 candidate solutions, as proven in Theorem 1 (see Materials and Methods for more details). Theorem 1 demonstrates that when the binding of $C_{1}$(or $C_{2}$) and R is stronger than that of $C_{2}$ (or $C_{1}$) and R, the unique biologically meaningful solution for $RC_{1}$ (or $RC_{2}$) is the smallest solution (i.e., $RC_{1}^{\left(1\right)}$ or $RC_{2}^{\left(1\right)}$) (i). This solution, initially expressed with complex terms, can be further simplified into real terms using the Lemma 1 to 4 in Text S1 (ii) (see Materials and Methods for more details). Finally, the formula for $RC_{1}$ (or $RC_{2}$) directly provides the formula for $RC_{2}$ (or $RC_{1}$) through the $RC_{1}$–$RC_{2}$ relation (iii). (C) This whole procedure provides solutions of both $\Psi_{1}\text{ and }\Psi_{1}$.

If $k_{m,1}=k_{m,2}=k_{m}$, then $\Psi_{1}\left(x\right)$ becomes quadratic equation asin Eqs. (11) and (12) is zero (similarly, $\Psi_{2}\left(x\right)$ as well). Therefore, the biologically meaningful solution of $\Psi_{1}\left(x\right)=0$ can be easily identified using the quadratic formula (see Case 1 of Text S1 for more details).

If $k_{m,1}<k_{m,2}$, Yan *et al*. [1] proved the existence of 3 distinct real roots of $\Psi_{1}\left(RC_{1}\right)=0,$ and the smallest root is the unique biologically meaningful solution. (We simplified Yan *et al*.’s proof of these lemmas in Case 2 of Text S1, using the signs of each coefficient of $\Psi_{1}\left(x\right)$ and $\Psi_{2}\left(x\right)$.) However, finding the smallest root of $\Psi_{1}\left(x\right)$ was still challenging. To overcome this, we derive an explicit expression that identifies the smallest root directly.

The main idea is that 3 real roots of $\Psi_{1}\left(x\right)$ correspond to the real-axis projections (horizontal components) of 3 complex numbers of equal modulus, separated by 120° in argument in the complex plane (Fig. S1). Each root is obtained by subtracting a positive multiple of the horizontal component of the corresponding complex number from a common constant, so the largest horizontal component corresponds to the smallest root. Since the 3 complex numbers are separated by 120°, exactly one of them necessarily falls within 60° of the positive horizontal axis (shaded wedge in Fig. S1), and this one, denoted P, has the largest horizontal component of the three, making it correspond to the smallest root. Writing the horizontal component of P in polar form gives the trigonometric expression that appears in Theorem 1 below (see Text S1 for more details of the derivation).

Theorem 1. Assume that $R_{\mathrm{tot}},k_{m,1},k_{m,2},T_{1}$, and $T_{2}$ are positive and that $k_{m,1}<k_{m,2}$. Then, $\Psi_{1}\left(RC_{1}\right)=a_{1}RC_{1}^{3}+b_{1}RC_{1}^{2}+c_{1}RC_{1}+d_{1}=0$ has 3 distinct real roots, and its smallest root is the unique biologically meaningful solution, given by

$$RC_{1}\left(T_{1},\;T_{2}\right)=-\frac{1}{3a_{1}}\left(b_{1}+\sqrt[{3}]{4}\;{\left(A^{2}+27a_{1}^{2}\Delta \right)}^{1/6}\text{cos}\left(\frac{1}{3}\text{atan}2\left(\sqrt{27a_{1}^{2}\Delta },\;A\right)\right)\right)$$ (17)

where$\Delta =b_{1}^{2}c_{1}^{2}-4a_{1}c_{1}^{3}-4b_{1}^{3}d_{1}-27a_{1}^{2}d_{1}^{2}+18a_{1}b_{1}c_{1}d_{1}$and $A=2b_{1}^{3}-9a_{1}b_{1}c_{1}+27a_{1}^{2}d_{1}$. Here, the convention $\text{atan}2\left(y,\;x\right)$ is used.

Using Theorem 1 and the relationship between $RC_{1}$ and $RC_{2}$ in Eqs. (9) and (10), the exact formula for $RC_{2}$ follows directly. If, $k_{m,1}>k_{m,2}$ the same procedure can be applied by switching the roles of $RC_{1}$ and $RC_{2}$.

### Computational speed of simulating the competitive qTMDD model with different methods

To compare 3 computational approaches solving a competitive qTMDD model—the exact formula, the bisection method, and the comparison method—we evaluated their computational efficiency using randomly sampled parameter sets (Fig. 3). Specifically, 100 independent parameter sets $\left(k_{m,1},\;k_{m,2},\;T_{1},\;T_{2},\;R_{\mathrm{tot}}\right)$ were sampled from $U\left(0,\;1000\right)$. For each parameter set, the total computation time required to calculate $RC_{1},RC_{2}$ 50,000 times using each method was measured in MATLAB (R2026a) with the “tic-toc” function to minimize the variability arising from single runs. The elapsed time was measured on a system with 8 GB of random access memory (RAM) and an Apple M2 processor.

**Fig. 3.**
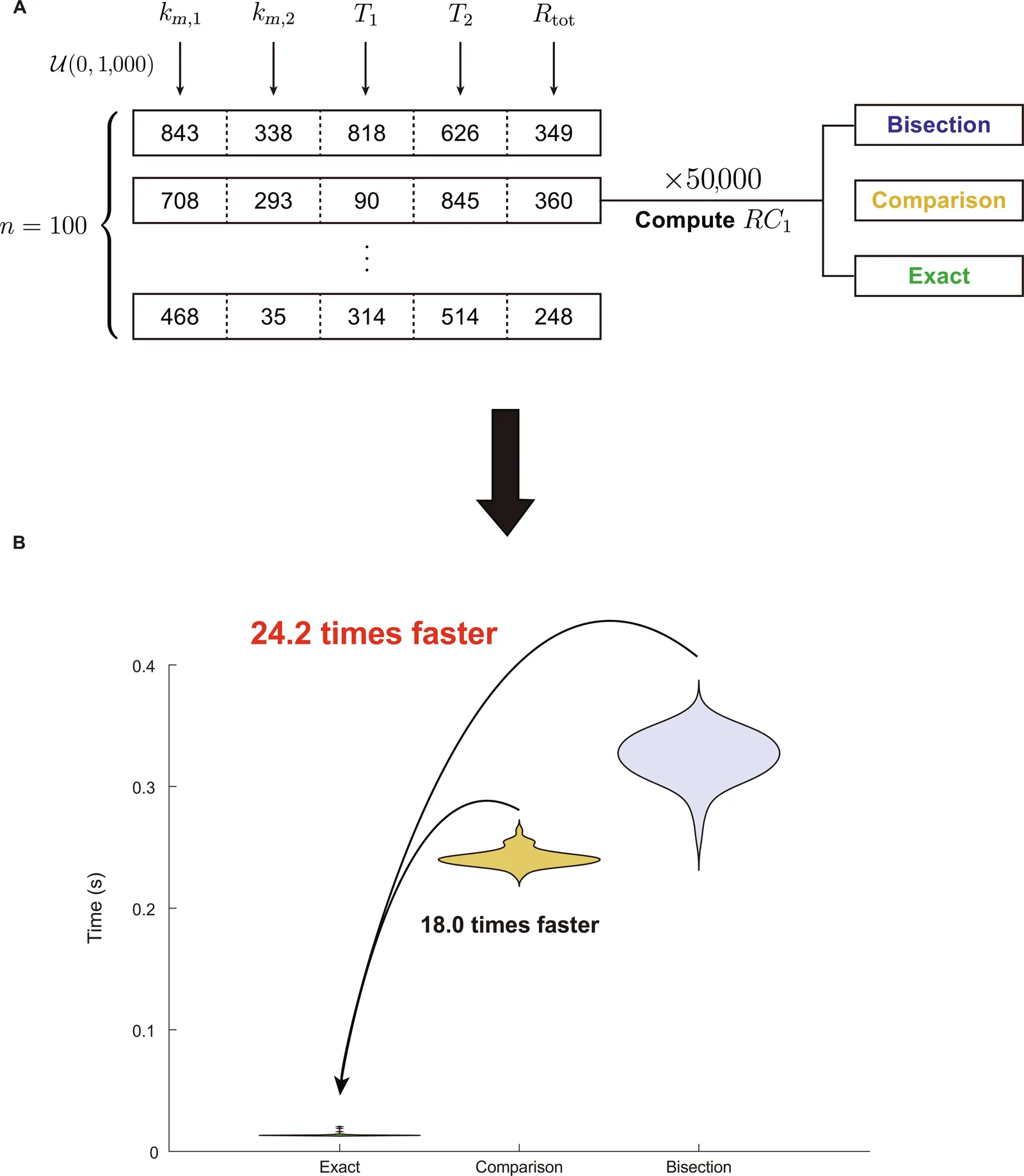
The exact formula provides the most efficient way of computing competitive qTMDD. (A) To compare the computation time in various parameter conditions, we generated 100 random parameter sets by randomly sampling each parameter from the uniform distribution: $U\left(0,\;1000\right)$. Then, we obtained a distribution of computation time by measuring the time it takes to compute $RC_{1}$ via tQSSA using each method (exact formula, comparison method, and bisection method) under each parameter set. (B) Computation of competitive qTMDD using the exact formula is 18.0 times faster than the comparison method and 24.2 times faster than the bisection method.

### Computational speed of Bayesian inference using competitive qTMDD with different methods

To compare the efficiency of 3 computational approaches solving competitive qTMDD with Bayesian inference, we measured the computational time of Markov Chain Monte Carlo (MCMC) iterations (Fig. 4). First, the stochastic time series data for $T_{1}$ and $T_{2}$ were generated using the Gillespie algorithm for the full competitive TMDD system with parameters $k_{\mathrm{el},1}=0,k_{\mathrm{on},1}=1,k_{\mathrm{off},1}=0.3,k_{\text{int},1}=1$(i.e., $k_{m,1}=1.3$) and $k_{\mathrm{el},2}=0,k_{\mathrm{on},2}=0.3,k_{\mathrm{off},2}=0.4,k_{\text{int},2}=2\;$ (i.e., $k_{m,2}=8$) with $T_{1}\left(0\right)=T_{2}\left(0\right)=20$ and $R_{\mathrm{tot}}=1$ or 100. Then, we assumed that $k_{\mathrm{el},1},k_{\mathrm{el},2},k_{m,2},k_{\text{int},1},$ and $k_{\text{int},2}$ are known and estimated $k_{m,1}\;$using Bayesian inference (Fig. 4). For this computational benchmark only, we set $k_{\mathrm{el},i}=0$ to isolate the target-mediated component and simplify the construction of the likelihood function; the general competitive qTMDD formulation retains the nonspecific elimination term.

**Fig. 4.**
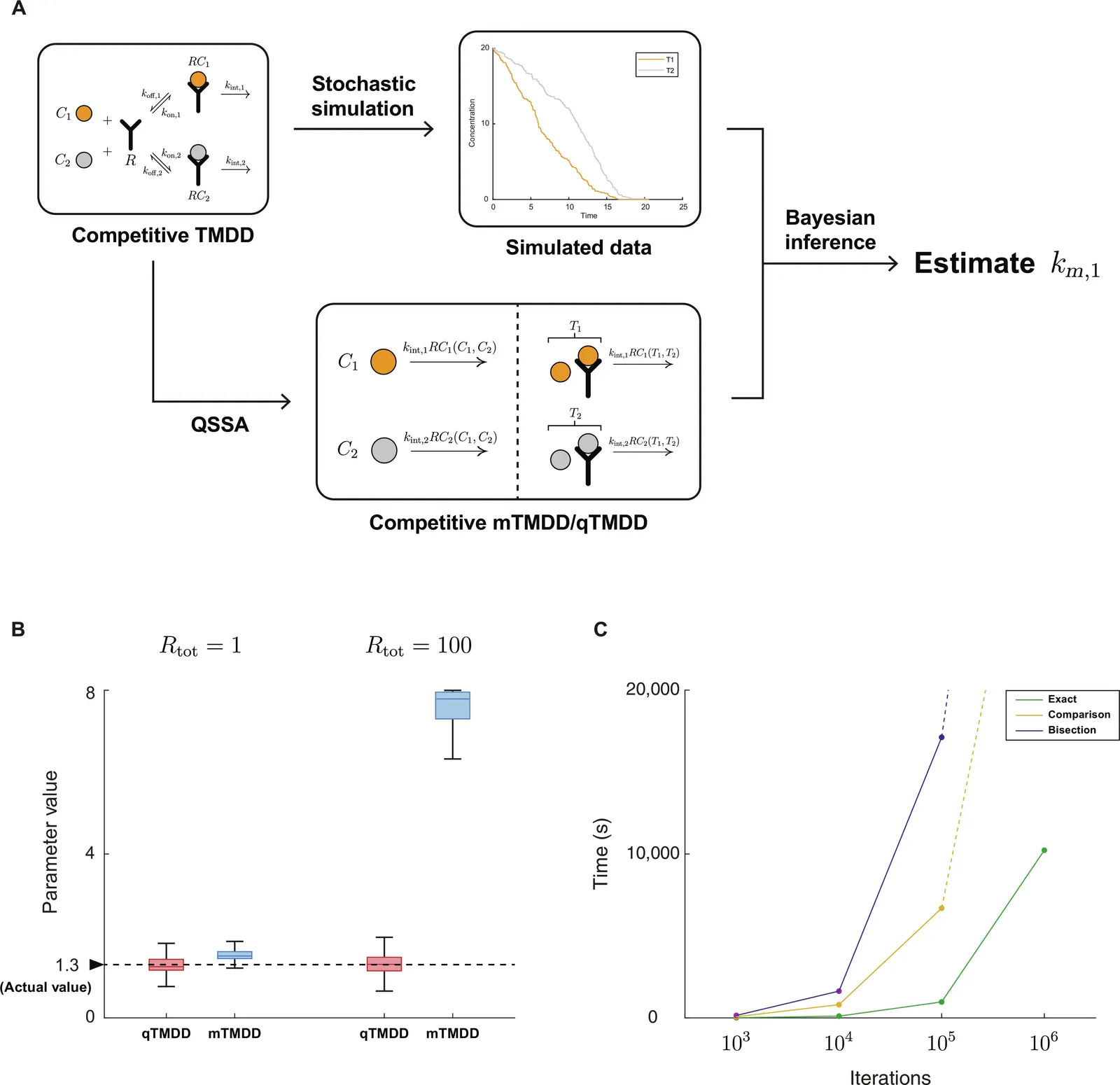
The exact formula provides an unbiased and efficient method of estimating parameters in a competitive qTMDD system. (A) We have obtained the simulated stochastic data using the Gillespie algorithm in a competitive target-mediated drug disposition (TMDD) system. To derive the likelihood of the simulated data, we have used both competitive mTMDD and qTMDD. Using the likelihood, we have estimated $k_{m,1}$ via Bayesian inference. (B) Parameter estimation using competitive qTMDD (red) provides unbiased inference results under all tested conditions. Meanwhile, competitive mTMDD (blue) provides biased estimation results, especially when total receptor concentration ($R_{\mathrm{tot}}$) is high. (C) Use of the exact formula (green) is the most efficient way to estimate parameters in competitive qTMDD, rather than the comparison method (yellow) or bisection method (blue).

The likelihood of the observed data (i.e., the time series data for $T_{1}$ and $T_{2}$) was constructed via Poisson process. Suppose the time-series data of total drug concentration is given as $D=\{\left(t_{1},\;T_{1,1},\;T_{2,1}\right),\left(t_{2},\;T_{1,2},\;T_{2,2}\right),\cdots ,\left(t_{n},\;T_{1,n},\;T_{2,n}\right)\}$. Since we assumed that $k_{\mathrm{el},i}=0$ in this computational benchmark, the rate of change of $T_{i}$ is $-k_{\text{int},i}RC_{i}$ (i=1,2); thus, for each k=1,2,⋯,n−1, the decrement of $T_{i}$ during $\left[t_{k},\;t_{k+1}\right]$ can be approximated by independent Poisson processes, each with rate $k_{\text{int},i}RC_{i}\left(k_{m,1},\;k_{m,2},\;T_{1,k},\;T_{2,k}\right)$ (i=1,2), where $RC_{i}\left(k_{m,1},\;k_{m,2},\;T_{1,k},\;T_{2,k}\right)$is the $RC_{i}$ value computed using competitive qTMDD.

From the previous assumption, for each k=1,2,⋯,n−1 and i=1,2:

$$t_{k+1}-t_{k}∼\text{Gamma}\left(T_{i,k}-T_{i,k+1},\;\frac{1}{k_{\text{int},i}RC_{i}\left(k_{m,1},k_{m,2},T_{1,k},T_{2,k}\right)}\right)$$ (18)

Therefore, the likelihood can be computed as:

$$P\left(D|\;k_{m,1}\right)\propto \prod_{k=1}^{n-1}\lambda_{1,k}^{n_{1,k}}\lambda_{2,k}^{n_{2,k}}{\left(t_{k+1}-t_{k}\right)}^{n_{1,k}+n_{2,k}-2}\exp\left(-\left(\lambda_{1,k}+\lambda_{2,k}\right)\left(t_{k+1}-t_{k}\right)\right)$$ (19)

where $\lambda_{i,k}=k_{\text{int},i}RC_{i}\left(k_{m,1},\;k_{m,2},\;T_{1,k},\;T_{2,k}\right)$, $n_{i,k}=T_{i,k}-T_{i,k+1}$ (i=1,2). By Bayes’ theorem, the posterior distribution is given as:

$$P\left(k_{m,1}|\;D\right)\propto P\left(D|\;k_{m,1}\right)P\left(k_{m,1}\right)$$ (20)

where $P\left(k_{m,1}\right)$ is the prior distribution of $k_{m,1}$, $\text{Uniform}\left(0,\;k_{m,2}\right)$. The time for estimating $k_{m,1}$ with $10^{3}$, $10^{4}$, $10^{5}$, $10^{6}$ MCMC iterations was measured for each of the 3 computational methods for solving qTMDD, and their distributions were reported (Fig. 4). The elapsed time was measured on a system with 8 GB of RAM and an M2 processor.

### Application of the exact formula of competitive qTMDD on real-world data

Two clinical cases were selected to evaluate approximation methods of competitive TMDD models. Case 1 involved competition between endogenous interleukin-1 (IL-1) and the exogenous drug anakinra at the IL-1 receptor, with 93 time–concentration observations from 8 individuals fitted to the model. Case 2 involved competition between endogenous IL-7 and the exogenous drug rhIL-7-hyFc at the IL-7 receptor, with 410 time–concentration observations from 24 individuals fitted. See [28,29] for detailed information. As described in the results section, *C*_0_ + *K*_M_ and *R*_tot_ should be compared, and we calculated (*C*_0_ + *K*_M_)/*R*_tot_ in each case for this purpose. In Case 1, this ratio was greater than 1 for the exogenous drug but less than 1 for the endogenous drug, suggesting that mTMDD would provide inaccurate approximations, whereas the exact formula of qTMDD would remain accurate. In Case 2, the ratio was greater than 1 for both the exogenous and the endogenous drugs, suggesting that both mTMDD and qTMDD would yield accurate approximations.

Within each case, the full TMDD, mTMDD, and qTMDD formulations were fitted to the real-world data. Previously established TMDD models describing drug–receptor interactions were adapted to incorporate competition between the exogenous drug and the endogenous ligand. Across the 3 models, only the structural approximation of receptor binding differed; all other components, including the interindividual variability structure, residual error model, and estimation settings, were kept the same. Case 1 applied this framework to drug–receptor competition only (Fig. S2A), whereas Case 2 additionally included neonatal Fc receptor (FcRn)-mediated recycling to capture systemic disposition (Fig. S2B). Parameter estimation was performed using NONMEM 7.5 and PsN 5.3.1. To avoid overparameterization, certain parameters were fixed to in vitro values reported previously: In Case 1, elimination rate of unbound endogenous compound (*k*_el_endo_), equilibrium constant of drug and receptor binding (*K*_SS_exo_), active concentration of total receptor (*R*_tot_), binding rate constant of drug–receptor (*k*_on_exo_), binding rate constant of endogenous compound–receptor (*k*_on_endo_), and dissociation rate constant of endogenous compound–receptor complex (*k*_off_endo_); in Case 2, *k*_on_exo_, dissociation rate constant of drug–receptor complex (*k*_off_exo_), *K*_SS_exo_, equilibrium constant of drug and FcRn binding (*K*_SS_FcRn_), *R*_tot_, active concentration of total FcRn (FcRn), uptake rate constant of drug from central to distribution space (*k*_uptake_), and recycling rate constant of drug from distribution space to central due to its binding with FcRn (*k*_recycle_). To minimize the influence of the initial values on the parameter estimation, we used an option that varied the initial estimates over a 90% range around the final model, and most parameter estimates differed by less than 2% in both cases. For both cases, receptor concentration was assumed constant, with receptor degradation set equal to the elimination rate of exogenous drug–receptor complex (*k*_deg_endo_ = *k*_int_exo_). In Case 2, the endogenous ligand concentration was also assumed constant, with *k*_deg_endo_ set equal to both *k*_int_exo_ and elimination rate of endogenous compound–receptor complex (*k*_int_endo_) ($k_{\text{deg}\_\text{endo}}=k_{\text{int}\_\text{exo}}=k_{\text{int}\_\text{endo}}$).

We compared the 3 fitted models in terms of estimation accuracy and computational efficiency. Estimation accuracy was defined as the degree of agreement with the parameter estimates from the full TMDD model, which was regarded as the reference standard. For this, we compared parameter estimates, objective function values (OFVs), residual-based diagnostic indices such as goodness-of-fit (GOF) plots, and simulation-based diagnostics such as visual predictive check (VPC) plots between approximation models and the full TMDD model. Computational efficiency was evaluated based on estimation and bootstrap times. Estimation time was measured by running the parameter estimation 10 times, with the initial condition set as the final parameter estimates shown in the Table S1. Then, its distribution was reported to account for variability due to random fluctuations. For bootstrap time, we resampled the original data 1,000 times, and for each resample, estimated parameters starting from the values in Table S1, reporting the distribution of the 1,000 estimation times. The elapsed time was measured on a system with 8 GB of RAM and an AMD Ryzen 7 5800H (3.2 GHz) processor for estimation, and on one with 256 GB of RAM and an AMD Ryzen Threadripper PRO 5965WX 24-core processor for bootstrap.

An additional analysis was conducted to assess whether the estimation results for Case 2 depended on the data-collection time point. We defined a dense grid by interpolating the early phase at 0.1-h spacing from 0 to 2 h and 0.5-h spacing from 2 to 25 h, simulated concentration–time profiles using the final full TMDD model, and then augmented the original dataset by adding only simulated observations at times not present in the original data; when a time overlapped, the original observation was retained. This procedure produced the densest set (+58). Starting from this +58 dataset, we obtained less-dense variants by sequentially removing early time points: +48 with 0.2-h spacing from 0 to 2 h, +42 with 0.5-h spacing from 0 to 2 h, and +17 with 1-h spacing from 0 to 25 h. Each of these datasets was analyzed with the final full TMDD, mTMDD, and qTMDD models, and the corresponding OFVs were compared to assess the impact of sampling density.

## Results

### The exact formula for competitive qTMDD is essential

TMDD, the process by which a drug binds to its target site (or receptor) [30], is typically modeled using a nonlinear system of ODEs (Fig. 1A, see Materials and Methods for details) [7,31–35]. This model has been used in a simplified form to enhance computational efficiency and reduce model parameters. Among various simplification methods, one of the most widely used methods is the QSSA (Fig. 1A) [11]. Applying sQSSA to the TMDD model yields the Michaelis–Menten TMDD model (mTMDD) by solving a simple linear equation for the drug–receptor complex (RC) (Fig. 1A (i)). In contrast, the QSS TMDD model (qTMDD), which is simplified with the tQSSA, involves solving a quadratic equation for RC and selecting the biologically meaningful solution (i.e., $0<RC<\min\left\{T,\;\;R_{\mathrm{tot}}\right\})$, where T is the total drug concentration and $R_{\mathrm{tot}}$ is the total receptor concentration among the 2 roots (Fig. 1A (ii)) (see Materials and Methods for details). Both mTMDD and qTMDD accurately approximate the TMDD when $R_{\mathrm{tot}}$ is low (Fig. 1B, left). In contrast, if $R_{\mathrm{tot}}$ is high (Fig. 1B, right), the accuracy of mTMDD deteriorates, while qTMDD remains accurate. In summary, for single-drug TMDD, the exact formula of both mTMDD and qTMDD can be derived, with qTMDD offering higher accuracy.

The competitive TMDD model, which describes the competition of 2 drugs that bind to an identical target, can also be simplified using QSSA (Fig. 1C). The competitive mTMDD simplified with sQSSA can be derived by solving a system of linear equations for the 2 drug–receptor complexes ($RC_{1},RC_{2}$) (Fig. 1C (iii)). However, this competitive mTMDD exhibits inaccurate approximation when total receptor concentrations are high (i.e., $C_{0}+K_{m}\ll R_{\mathrm{tot}}$; Figs. S3 and S4), similar to the single-drug case. Given that single-drug TMDD was more accurately approximated with tQSSA, its application to the competitive TMDD model, namely, competitive qTMDD, is essential. Deriving the competitive qTMDD requires solving a cubic equation for $RC_{1}$ and $RC_{2}$ (Fig. 1C (iv)). This cubic equation yields 3 roots; however, identifying the biologically meaningful solution among them is nontrivial due to the presence of complex terms (Fig. 1C (v)). To address this, a previous study [1] proved that the smallest of the 3 real roots is the unique biologically meaningful solution but did not provide a method to identify the smallest root. Consequently, the exact formula of competitive qTMDD remains unknown even while it provides a more accurate approximation than competitive mTMDD (Figs. S3 to S5).

As the exact formula of competitive qTMDD is unknown, its simulation has relied on numerical approximation schemes solving cubic equations (Fig. 1D and E). For instance, the bisection method (Fig. 1D), which finds the approximate root in a specific interval, has been used (see Materials and Methods for more details). Starting from the interval where the biologically meaningful solution is guaranteed to exist (i.e., $0<\mathrm{RC}<\min\left\{T,R_{\mathrm{tot}}\right\}$), the solution is approximated by bisecting the intervals into halves (Fig. 1D, left). However, reducing the error of this approximation as much as desired requires numerous iterations, leading to long computation times. Indeed, the computation time (i.e., number of iterations needed to approximate the solution within the error bound) increases steeply as the error bound decreases (Fig. 1D, right). Therefore, using the bisection method to compute competitive qTMDD is highly inefficient.

As an alternative, the comparison method (Fig. 1E), which calculates all 3 roots of the cubic equation using the cubic formula involving complex numbers, and checks whether each solution is biologically meaningful, has also been used (see Materials and Methods for more details). However, the major limitation of this method is its reliance on complex number arithmetic, which is not compatible with real valued data by the nonlinear mixed effect modeling (NONMEM) software, widely implemented in the field of pharmacometrics. While this issue can be solved by using another numerical approximation algorithm (see Materials and Methods for more details), this algorithm requires massive computation. As a result, the comparison method is also impractical, highlighting the urgent need for an exact formula of competitive qTMDD.

### We derived the exact formula of competitive qTMDD

To derive the exact formula of competitive qTMDD, a previous study [1] showed that the biologically meaningful solution is the smallest root among the 3 roots of the cubic equation (see Lemma 1 to 4 in Text S1). However, identifying the smallest root analytically proved challenging. As a result, despite the theoretical insights, the computation of competitive qTMDD continued to rely on numerical approaches such as the bisection or comparison methods.

To overcome this limitation, we found the exact formula of competitive qTMDD by explicitly identifying the smallest root among the 3 roots (Fig. 2A; see Materials and Methods for more details). We first derived the exact form of the concentration of drug–receptor complex associated with the stronger binding affinity (Fig. 2B (i); see Theorem 1 for more details). For instance, if drug $C_{1}$ has a stronger binding affinity than drug $C_{2}$, then the smallest root of the cubic equation for $RC_{1}$ can be analytically obtained (Fig. 2B (i)). Then, this was reformulated to contain only real-valued terms (Fig. 2B (ii); see Theorem 1). This formula of RC with stronger binding affinity was substituted into the RC relationship equations derived from the QSSA equations (Fig. 2B (iii); see Eqs. (9), (10)), to calculate the exact formula of RC with the weaker binding affinity. Taken together, we derived the exact formula for both drug–receptor complexes ($RC_{1}$ and $RC_{2}$), obtaining the exact formula of competitive qTMDD.

### The exact formula provides efficient computation of competitive qTMDD

We evaluated whether the exact formula enables faster computation of competitive qTMDD compared to previous methods—the bisection method and the comparison method. To do this, we measured the time required to compute $RC_{1}\;\text{and}\;RC_{2}$ (i.e., the roots of the cubic equation in Fig. 1C(iv)) under various parameter conditions (Fig. 3A). In particular, 100 parameter combinations were generated by independently sampling each of the 5 different parameters ($k_{m,1},k_{m,2},T_{1},T_{2},R_{\mathrm{tot}}$) from the uniform distribution $U\left(0,\;1000\right)$. For each parameter combination, $RC_{1}\;\text{and}\;RC_{2}$ were computed 50,000 times using each of the 3 methods: the exact formula, the bisection method, and the comparison method (see Materials and Methods for more details). This repetition was performed to ensure stable measurement of computation time, minimizing the effect of variability from single runs. The total computation times were visualized using violin plots (Fig. 3B).

The results show that the exact formula substantially improves computational efficiency (Fig. 3B). Specifically, it achieved a 24.2-fold speedup over the bisection method and an 18.0-fold speedup over the comparison method (Fig. 3B; see Materials and Methods for more details). Even when compared with more advanced numerical root-finding approaches, such as the Newton–Raphson method, the exact formula remains substantially faster (Fig. S6). In addition, the Newton–Raphson method frequently converges to incorrect roots (Fig. S6). Taken together, these results demonstrate that the exact formula significantly enhances the practicality of applying competitive qTMDD by drastically reducing computation time.

### The exact formula of competitive qTMDD allows efficient and unbiased Bayesian inference for competitive TMDD

The rapid computation of competitive qTMDD with the exact formula opens the door to its use in Bayesian inference, which was previously impractical due to the high cost of iterative computations. To demonstrate this potential, we used Bayesian inference to estimate the first Michaelis constant in competitive TMDD ($k_{m,1}$) (Fig. 4A; see Materials and Methods for more details). We first generated the synthetic data of the full competitive TMDD model with a Gillespie algorithm (see Materials and Methods for more details). This synthetic data was then used to estimate $k_{m,1}$ by fitting 2 reduced models: competitive mTMDD and qTMDD.

While $k_{m,1}$ was accurately estimated using competitive qTMDD (Fig. 4B), inference based on the competitive mTMDD overestimated the value of $k_{m,1}$ under high receptor concentration conditions (Fig. 4B, right). This indicates that the use of competitive qTMDD is crucial for accurate parameter inference. However, applying the competitive qTMDD with previous numerical methods requires a substantial computational cost (Fig. 4C). In particular, 1.8 and 5.6 h were required when using the comparison method and the bisection method, respectively, to complete $10^{5}\;$ iterations of MCMC sampling. In contrast, the explicit formula reduced this time to approximately 0.25 h. These results highlight that the explicit formula for competitive qTMDD is not only essential for accuracy but also enables practical use of Bayesian inference by significantly reducing computation time.

### The exact formula of competitive qTMDD estimates pharmacological parameters accurately and efficiently with real-world clinical data

Fast and accurate parameter estimation using the exact formula of the competitive qTMDD model shows its potential for estimating parameters in real-world clinical data. To investigate this potential, we estimated the pharmacological parameters by fitting full TMDD, mTMDD, and the exact formula of qTMDD to 2 representative real-world competitive TMDD cases, in which an endogenous and an exogenous substance compete for receptor binding: Case 1 for anakinra and Case 2 for rhIL-7-hyFc. In Case 2, additional parameters for FcRn-mediated recycling and intramuscular dosing were included to account for the pharmacokinetic properties specific to rhIL-7-hyFc (see Materials and Methods for more details).

In Case 1, the ratio $\left(C_{0}+K_{M}\right)/R_{\mathrm{tot}}$ for the endogenous drug is less than 1; thus, we expected that the mTMDD approximation would be invalid, whereas the qTMDD model would remain valid. Indeed, estimates of *V*_c_, *k*_a_SC_, IIV_*V*_c_, and IIV_*k*_a_ from the mTMDD model differed from those estimated by full TMDD, whereas qTMDD yielded parameter values close to those of the full TMDD model (Fig. 5A), despite comparable GOF (GOF plots; Fig. S7) and predictive performance (VPC plots; Fig. S8). For example, the estimated *V*_c_ was 52.9 when using qTMDD and full TMDD, while mTMDD yielded 22.2 (Table S1).

**Fig. 5.**
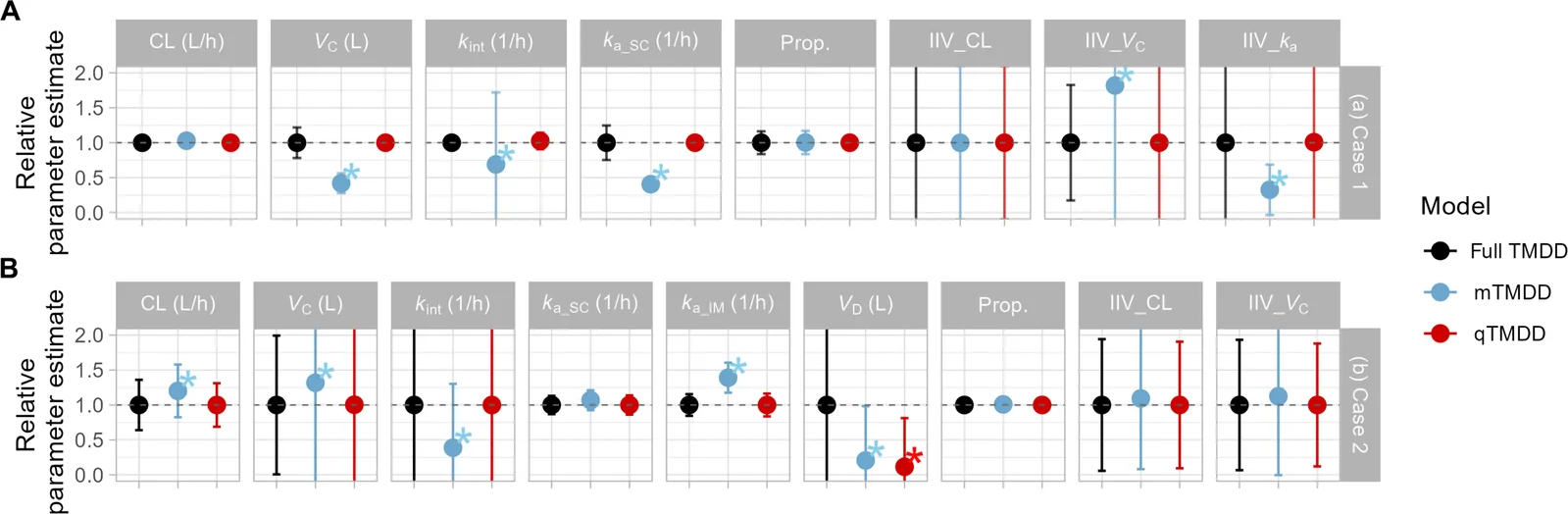
The competitive qTMDD yields parameter estimates similar to those of full competitive target-mediated drug disposition (TMDD) in real-world cases. (A) Plots of relative parameter estimates in Case 1 using full TMDD (black), mTMDD (blue), and qTMDD (red). Full TMDD and qTMDD yield similar estimates for *V*_c_, *k*_int_, *k*_a_SC_, IIV_*V*_c,_, and IIV_*k*_a_. (B) Plots of relative parameter estimates in Case 2 using full TMDD (black), mTMDD (blue), and qTMDD (red). Full TMDD and qTMDD yield similar estimates for CL, *V*_c_, *k*_int,_, and *k*_a_IM_. CL, apparent clearance of drug at central compartment; *V*_c_, apparent volume of distribution of drug and endogenous compound at central compartment; *k*_a_, absorption rate constant of drug from injection site (IM for intramuscular injection, SC for subcutaneous injection); *k*_int_, elimination rate of drug–receptor complex; Prop., proportional error; IIV, interindividual variability. Asterisks (*) indicate parameters whose estimates in the approximation model deviated from those of the full TMDD model. Dots indicate relative parameter estimates, and error bars represent the corresponding 95% confidence intervals (CIs), calculated as CI = (Estimate/baseline) ± 1.96 * (Standard error / baseline). The dashed line at 1 indicates the full TMDD reference.

In Case 2, the ratio $\left(C_{0}+K_{M}\right)/R_{\mathrm{tot}}$ is greater than 1 for both the endogenous and exogenous drugs. Thus, we expected that both the mTMDD and qTMDD models would be valid. However, unexpectedly, estimates of CL, *V*_c_, *k*_a_IM_, *k*_a_SC_, *k*_int_, IIV_CL, and IIV_*V*_c_ from the mTMDD model still deviated from those of full TMDD, while qTMDD yielded similar estimates (Fig. 5B). Furthermore, the OFV of the mTMDD model was lower than that of the qTMDD model (Table S1), while GOF plots and VPCs were comparable.

To investigate the reason for this discrepancy between the validity condition and the parameter estimation results from the mTMDD model in Case 2, we generated a denser dataset by interpolating the original data using the fitted full TMDD model and compared the parameter estimates from the original dataset with sparse early-phase sampling (410 observations) and the interpolated dense dataset (1,440 observations) (Fig. 6). With the denser dataset, parameter estimates from the mTMDD model became closely aligned with those from the full TMDD model (Fig. 6A and Table S2). In particular, the OFV value from mTMDD—initially low with the original dataset—increased progressively as more interpolated data were added (Fig. 6B).

**Fig. 6.**
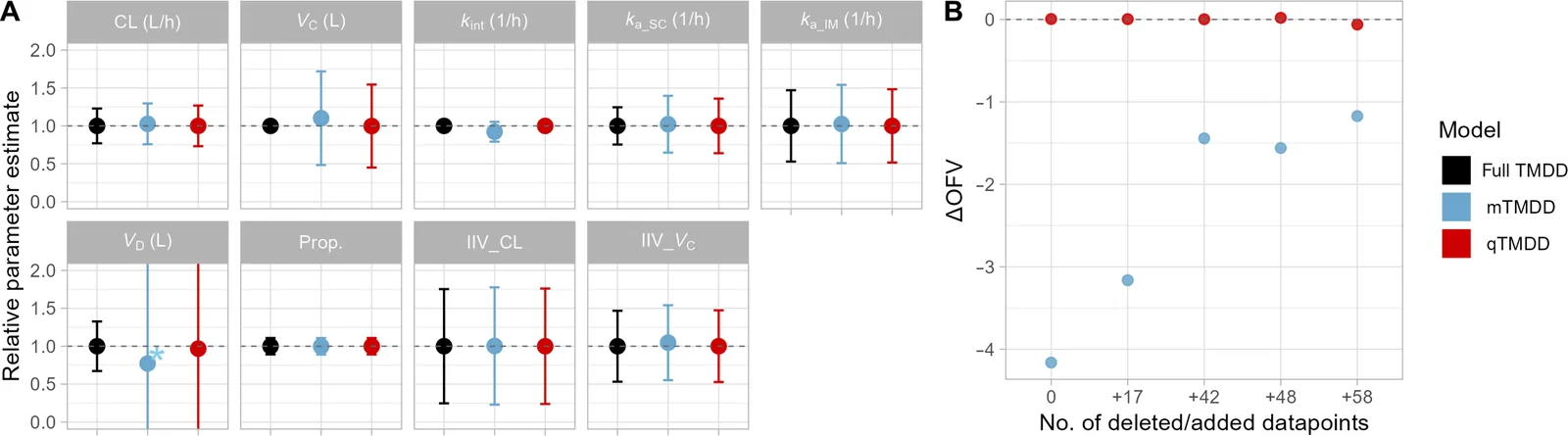
qTMDD yields parameter estimates similar to those of full competitive target-mediated drug disposition (TMDD) across different sampling densities. (A) Plots of relative parameter estimates in densified data using full TMDD (black), mTMDD (blue), and qTMDD (red). mTMDD showed a smaller difference from full TMDD compared to the original data. Refer to Fig. 5 for parameter definitions. (B) Effect of adding data points on the objective function value (OFV). For mTMDD, the OFV was 4.162 lower than that of the full TMDD at baseline; this difference narrows as the dataset is densified. ΔOFV is defined as OFV of the approximation model − OFV of the full TMDD model. See Materials and Methods for details of the dataset densification/reduction procedure.

These results indicate that limited temporal sampling, particularly during the early postdose phase, allowed compensatory changes in the mTMDD parameter estimates, yielding a model-specific optimum that differed from the full TMDD estimates. As sampling density increased, the full concentration–time profile became more strongly constrained and the mTMDD estimates progressively approached those obtained with the full TMDD model. Thus, even when the conventional validity condition is satisfied, small approximation errors during transient phases may lead to biased parameter estimates when informative observations are limited.

Next, we investigated the computational advantage of qTMDD compared to the full TMDD model. To evaluate computational efficiency, we calculated the time required for estimating parameters over 10 repeated runs (estimation time) and the time required for estimating 1,000 resampled datasets with successful convergence to account for dataset-dependent variability (bootstrap time). For both analyses, estimation was initialized using the final parameter estimates of the converged model (see Materials and Methods for more details). The results show that both estimation time and bootstrap time are shorter when using qTMDD than when using full TMDD (Fig. 7). In Case 1, qTMDD reduced estimation time by approximately 72.0% and bootstrap time by 56.7% relative to the full TMDD model (Fig. 7). In Case 2, the corresponding reductions were 8.2% and 68.9%, respectively (Fig. 7). These results highlight that qTMDD offers a practical and more efficient alternative to full TMDD for real-world pharmacokinetic analyses.

**Fig. 7.**
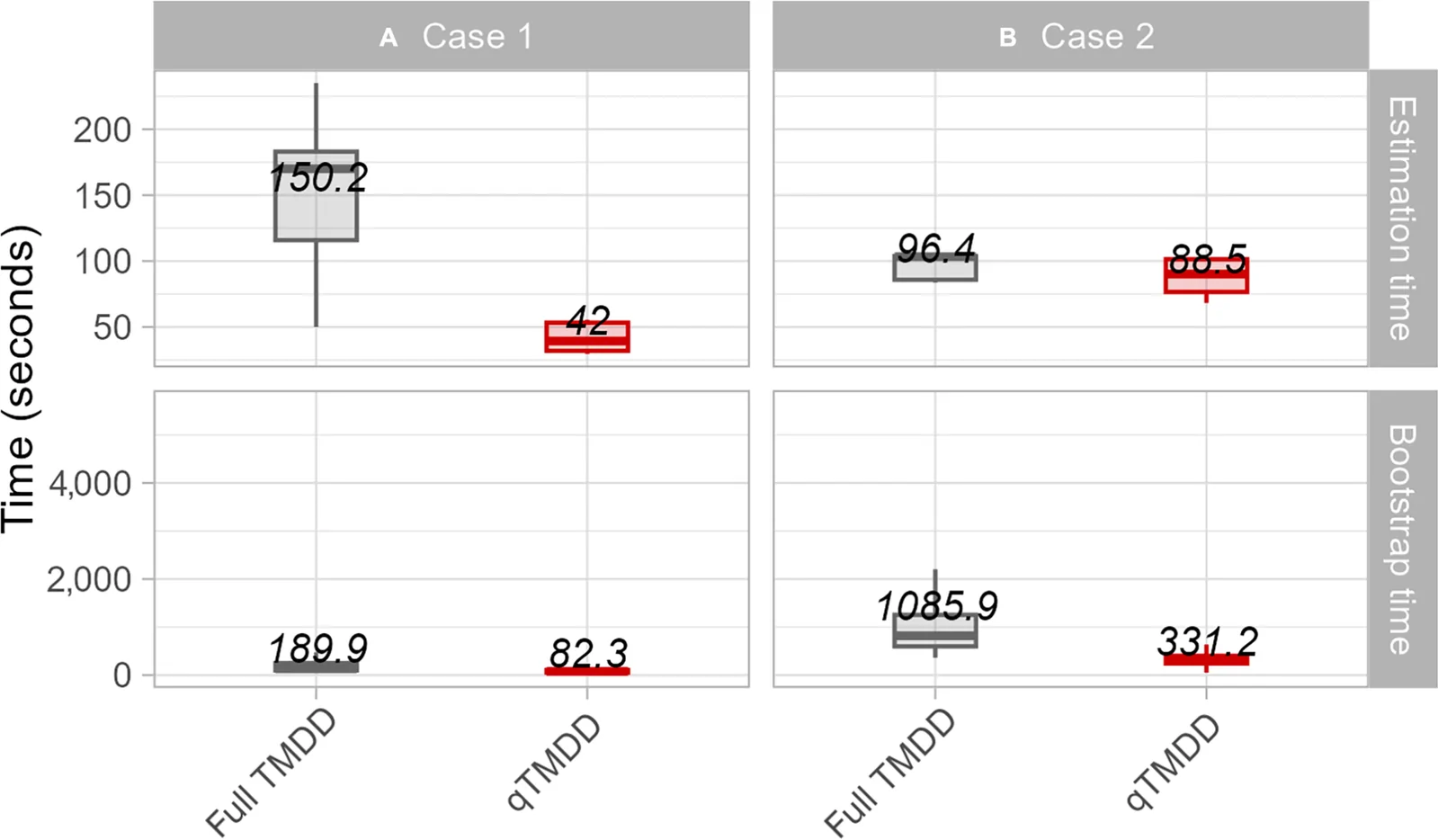
Competitive qTMDD enables faster parameter estimation than full competitive target-mediated drug disposition (TMDD). Boxplots of estimation time (top) and bootstrap time (bottom) in Case 1 (A) and Case 2 (B) using full TMDD (gray) and qTMDD (red). Estimation times were obtained from 10 repeated runs of the final convergent model, while bootstrap times were obtained from 1,000 resampled datasets (successful runs only). For both estimation time and bootstrap time, qTMDD is faster than full TMDD. In the box plot, the line represents the median, the box represents the interquartile range, and the whiskers extend to values within 1.5 times the interquartile range.

## Discussion

Although competitive qTMDD is essential to accurately approximate the competitive TMDD system, its derivation requires solving a cubic equation (Fig. 1C). While this can be done by using a cubic formula, the biologically meaningful solution among the 3 roots of the cubic equation was unknown, leading to the use of inefficient approximation methods such as the bisection or the comparison method (Fig. 1D and E). To address this problem, we derived the unique biologically meaningful solution, the exact formula of competitive qTMDD, by utilizing the analytic properties of cubic functions and the geometric properties of the complex plane (Fig. 2). This exact formula enabled competitive qTMDD simulations that were approximately 18 times faster than previous approximation methods (Fig. 3). These rapid simulations enabled Bayesian inference using competitive qTMDD, and applying this to the simulation data resulted in more accurate and unbiased pharmacological parameter estimation than competitive mTMDD (Fig. 4). Furthermore, the use of the exact formula of competitive qTMDD in real-world data enabled faster parameter estimation while yielding parameters similar to those estimated by the full TMDD model (Fig. 5). These results demonstrate that the exact formula of competitive qTMDD allows accurate and efficient pharmacological parameter estimation.

Parameter estimation using competitive qTMDD is likely to play an important role in pharmacometrics. With the rapid growth in biologics development, the use of TMDD models has expanded quickly. Although competitive target binding is often overlooked in clinical pharmacokinetic analyses, it can be important in specific clinical settings. Given this context, an exact formula for competitive qTMDD—one that accurately approximates the full competitive TMDD model while preserving computational efficiency—offers clear advantages for practical pharmacometric analyses. In particular, this accurate approximation includes robustness to limited sampling density: Even when the theoretical validity conditions for mTMDD were satisfied, a small discrepancy remained during the early postdose phase (Fig. S9), and lower sampling density allowed parameter compensation, resulting in mTMDD parameter estimates that were different from those of the full TMDD model, whereas competitive qTMDD remained closely aligned with the full TMDD model in both original and densified datasets (Fig. 6). This robustness is important in clinical pharmacokinetic studies, where the number and timing of samples are constrained by ethical and logistical considerations.

An additional practical advantage of competitive qTMDD is the reduction in the number of independently estimable quantities. In the full TMDD model, microscopic binding parameters such as *k*_on_, *k*_off_, and *k*_int_ enter separately and may be difficult to identify from conventional pharmacokinetic observations alone. Under the tQSSA, these parameters are partially represented through composite quantities such as *K*_M_ = (*k*_off_ + *k*_int_)/*k*_on_, reducing the effective dimensionality of the estimation problem. However, this reduction does not eliminate identifiability limitations arising from incomplete observability. In the present clinical datasets, only unbound drug concentrations were measured, and several mechanistic parameters therefore remained weakly informed and had to be fixed using literature or in vitro values. Nevertheless, the parameter-correlation structure and profile likelihoods of qTMDD closely matched those of the full TMDD model, indicating that the qTMDD approximation does not introduce substantial additional parameter interdependence (Figs. S10 and S11 and Table S6). In terms of implementation, previous formulations of competitive qTMDD relied on complex-valued solutions or decision trees, which made it difficult to implement them in standard tools such as NONMEM. In contrast, our exact formula can be directly implemented in conventional PopPK/PD workflows as demonstrated by its successful use and numerical robustness in NONMEM (Table S5). This enables direct estimation and simulation within population pharmacokinetic software, allowing model-based dose selection and scenario prediction to be immediately applicable in real clinical settings where competitive interactions are present, while also providing a basis for future integration with downstream pharmacodynamic models.

Our comparison of sampling densities revealed a clear asymmetry between the 2 reduced models: mTMDD recovered the full TMDD estimates well when observations were dense but yielded systematically biased parameters under the sparse schedule, even though the theoretical validity condition $\left(C_{0}+K_{m}\gg R\_\mathrm{tot}\right)$ was satisfied throughout (Fig. 6). This arises from the fact that mTMDD retains a small but systematic deviation confined to the early postdose phase, in which the rapid initial binding transient is not fully captured. Critically, satisfaction of the validity condition does not preclude such a deviation: The condition governs the accuracy of the approximation only after the binding reaction has relaxed to QSS [17,36], and by construction it says nothing about the brief initial period during which that state is being established. This is not specific to the competitive TMDD setting examined here. mTMDD is the pharmacokinetic counterpart of the sQSSA of Michaelis–Menten kinetics, whose validity conditions likewise apply only outside the initial transient layer, so that the approximation is known to break down during the period preceding establishment of the QSS; total-concentration-based reductions such as the tQSSA—of which qTMDD is the direct analog—remain accurate across this phase and over a substantially wider parameter range [21,22]. It would therefore be of interest to examine whether the sampling-dependent estimation uncertainty observed here generalizes to other sQSSA-based reductions used in systems pharmacology and enzyme kinetics and to establish more generally which features of the experimental design determine when such reductions can be safely used for parameter estimation.

Beyond the TMDD systems, the tQSSA-based reduction has also improved the accuracy of drug metabolism predictions, including applications in predicting hepatic intrinsic clearance and assessing drug–drug interactions mediated by the cytochrome P450 (CYP) induction [37–40]. While these applications are grounded on a single-enzyme kinetics where tQSSA derivation is straightforward, extending this framework to systems involving multiple enzymes results in a complex mathematical problem requiring the solution of a cubic equation—analogous to the competitive TMDD case addressed in this study [27,41]. We anticipate that our approach can be similarly applied to derive tQSSA reductions for multiple-enzyme drug metabolic systems, and investigating this potential with real-world data remains a promising avenue for future work.

Another important advantage of adopting the tQSSA-based reduction is that it resolves the structural limitations of sQSSA: Satisfying the validity condition does not guarantee accurate parameter estimation, as shown in Fig. 5B. In particular, Choi *et al*. [42,43] addressed this structural identifiability issue by leveraging the wide validity of tQSSA to integrate data across various experimental conditions and implement a sequential experimental design. In this framework, parameter estimates derived from an initial dataset are utilized to determine the optimal initial concentrations for subsequent experiments, thereby maximizing the precision of the final estimation. Adopting this tQSSA-driven sequential strategy for both competitive TMDD systems and multiple-enzyme drug metabolic systems would allow for a more accurate determination of kinetic parameters in complex competitive environments, representing a critical direction for future research.

Despite the advantages of competitive qTMDD, the high complexity of the exact formula makes its intuitive interpretation difficult (see Theorem 1 in Materials and Methods). Similar to the simplification of qTMDD to pTMDD in a noncompetitive case [11], future work on simplifying the competitive qTMDD formulation will be important for broader adoption. Another potential direction of future work involves extending the framework to scenarios in which 3 or more drugs compete for a single receptor. In such situations, generalizing the derivation used for the 2-drug case to a multidrug competitive system will be necessary to obtain an exact competitive qTMDD formula. In addition, extending our approach to a stochastic and spatially distributed system represents a promising direction. Notably, stochastic systems necessitate different validity conditions for QSSA compared to deterministic ones [44–47], and spatial heterogeneity is known to alter the validity of the Michaelis–Menten rate law and QSSA in partial differential equation models versus well-mixed ODE models [48,49]. Given this significant impact of stochasticity and spatial heterogeneity on QSSA, extending our framework to encompass these conditions would broaden its applicability to a wider range of realistic biological scenarios.

The present work derives an exact analytical solution to the competitive tQSSA within the modeling assumptions established in the literature on which it builds [1,7]. These assumptions, however, may limit the applicability of the framework to real TMDD systems: The total receptor concentration is taken to be constant, and free-receptor internalization and turnover—processes central to TMDD clearance dynamics—are omitted. Moreover, by imposing a QSS algebraic relation, the tQSSA does not resolve the fast boundary and transition layers of the dynamics, such as the rapid binding phase immediately after dosing.

Addressing these limitations would require moving beyond a single reduced model. Singular-perturbation techniques, in particular the method of matched asymptotic expansions—which analyze the system separately on each characteristic time scale and match the resulting solutions across adjacent phases—are well suited to capturing both the fast transient layers and the slow elimination dynamics within one consistent theory [7–10].

A promising direction for future work is therefore to combine the two: embedding the exact closed-form solution developed here within a broader multiple-time-scale treatment that additionally resolves the fast layers and relaxes the constant-receptor assumption. This would retain the computational efficiency of the exact tQSSA solution within the QSS regime while extending the range of dynamics that can be faithfully represented.

There are also limitations when applying competitive qTMDD to real-world data in our analysis. In particular, time-varying physiological changes such as changes in receptor expression were not explicitly modeled. In addition, in the dataset analyzed here, exogenous drugs are administered at much higher concentrations than endogenous substances, resulting in limited information about endogenous dynamics. Conducting similar analyses in settings where exogenous and endogenous concentrations are of comparable magnitude would help further validate the utility of competitive qTMDD across a broader range of clinically relevant scenarios. In addition, future studies could extend the competitive qTMDD framework by coupling receptor-level pharmacokinetic interactions with downstream pharmacodynamic models that describe concentration–effect or receptor occupancy–response relationships. Such an extension would enable the framework to investigate how competitive target binding translates into synergistic, additive, or antagonistic pharmacodynamic effects of drug combinations.

## Data Availability

The real-world data for anakinra and rhIL-7-hyFc are presented in Tables S3 and S4, respectively. The simulation codes underlying this work are provided as a ZIP file “qTMDD.zip”, File S1. The NONMEM codes are available in Text S2.
