# Supplementary material for: Exact Formula of the Total Quasi-Steady State Approximation in Competitive Target-Mediated Drug Disposition
Source: Comput Struct Biotechnol J. 2026 Oct 8;35(1):0223. doi: 10.34133/csbj.0223 (PMC13646460; doi:10.34133/csbj.0223)
Supplement: Supplementary file 1 [file csbj.0223.f1.zip › S1 Fig.pdf]

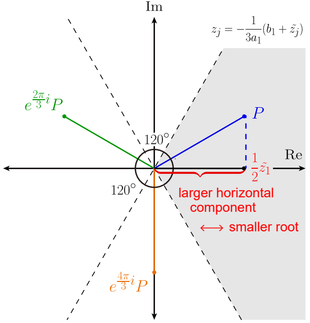

Im
$z_j = -\frac{1}{3a_1}(b_1 + \tilde{z}_j)$
$e^{\frac{2\pi}{3}i}P$
P
120°
Re
$\frac{1}{2}\tilde{z}_1$
120°
larger horizontal component
⟷ smaller root
$e^{\frac{4\pi}{3}i}P$
