# Supplementary material for: Exact Formula of the Total Quasi-Steady State Approximation in Competitive Target-Mediated Drug Disposition
Source: Comput Struct Biotechnol J. 2026 Oct 8;35(1):0223. doi: 10.34133/csbj.0223 (PMC13646460; doi:10.34133/csbj.0223)
Supplement: Supplementary file 1 [file csbj.0223.f1.zip › S1 Table.pdf]

Table S1. Parameter estimate result, OFV values and elapsed time

**(a) Case 1:** $(C_0+K_M)/R_{tot\_exo}$ = 23.86, $(C_0+K_M)/R_{tot\_endo}$ = 0.04						
	**Full TMDD**		**mTMDD**		**qTMDD**	
OFV	358.847		360.524		358.879	
deltaOFV	-		1.677		0.032	
Parameter estimates (unit)						
CL (L/h)	9.69	(2.2%)	9.97	(4.0%)	9.69	(4.2%)
V_C (L)	52.9	(2.2%)	22.2	(17.2%)	52.9	(7.2%)
k_a (1)	0.448	(1.0%)	0.182	(10.4%)	0.448	(6.3%)
k_{int} (1/h)	0.0717	(4.4%)	0.0494	(76.3%)	0.0735	(4.7%)
Inter-individual variability (variance)						
CL	0.0108	(82.4%)	0.0108	(55.8%)	0.0108	(55.8%)
V_c	0.113	(47.3%)	0.206	(58.7%)	0.113	(56.0%)
CL-V_c cov	0.001	-	0.0062	-	0.0007	-
k_a	0.254	(74.8%)	0.083	(56.3%)	0.256	(59.4%)
Residual error (%)						
Proportional	11.6	(1.8%)	11.6	(8.5%)	11.6	(5.0%)
Elapsed time (s)						
Estimation	150.18	(53.78)	26.69	(7.68)	41.95	(10.86)
Bootstrap	189.89	(135.88)	59.14	(47.44)	82.27	(46.08)
**(b) Case 2:** $(C_0+K_M)/R_{tot\_exo}$ = 21.91, $(C_0+K_M)/R_{tot\_endo}$ = 55.88						
	**Full TMDD**		**mTMDD**		**qTMDD**	
OFV	1642.644		1638.482		1642.65	
deltaOFV	-		-4.162		0.006	
Parameter estimates (unit)						
CL (L/h)	13	(18.4%)	15.6	(16.0%)	13	(15.9%)
V_C (L)	27.4	(52.2%)	36	(62.2%)	27.4	(57.7%)
k_a (1/h)	0.00775 (im)<br>0.00239 (sc)	(8.0%)<br>(6.5%)	0.0108<br>0.00255	(7.9%)<br>(6.8%)	0.00774 (im)<br>0.00239 (sc)	(8.3%)<br>(7.0%)
k_{int} (1/h)	1.17	(64.1%)	0.455	(120.0%)	1.17	(61.3%)
V_D (L)	0.00297	(141.8%)	0.000609	(192.1%)	0.00034	(308.8%)
k_{on_endo} (1/(pM*h))	0.077	(370.8%)				
Inter-individual variability (variance)						
CL	0.176	(50.1%)	0.192	(47.3%)	0.176	(46.2%)
V_c	1.68	(44.2%)	1.89	(51.2%)	1.68	(44.9%)
Residual error (%)						
Proportional	35.7	(2.7%)	35.7	(2.9%)	35.7	(2.9%)
Elapsed time (s)						

Estimation	96.39	(9.45)	33.2	(8.4)	88.48	(13.86)
Bootstrap	545.05	(327.16)	217.21	(118.58)	397.38	(222.59)

CL: Apparent clearance of drug at central compartment, V_c: Apparent volume of distribution of drug and endogenous compound at central compartment, k_a: Absorption rate constant of drug from injection site (im for intramuscular injection, sc for subcutaneous injection), k_{int}: elimination rate of drug-receptor complex, k_{syn}: synthesis rate of endogenous compound and V_D: Apparent volume of distribution of drug at FcRn-mediated recycling site. Values are point estimates; numbers in parentheses are the relative standard error for estimation results (RSE, %) and standard deviations for elapsed time (seconds).
