# Supplementary material for: Exact Formula of the Total Quasi-Steady State Approximation in Competitive Target-Mediated Drug Disposition
Source: Comput Struct Biotechnol J. 2026 Oct 8;35(1):0223. doi: 10.34133/csbj.0223 (PMC13646460; doi:10.34133/csbj.0223)
Supplement: Supplementary file 1 [file csbj.0223.f1.zip › S1 Text.pdf]

1 Biologically Meaningful Solution for Competitive qTMDD

The equations for competitive qTMDD are given as

$$\begin{aligned}
\Psi_1(x) &= a_1x^3 + b_1x^2 + c_1x + d_1 \\
a_1 &= k_{m,2} - k_{m,1} \\
b_1 &= -(k_{m,2} - k_{m,1})(R_{\text{tot}} + T_1 + k_{m,1}) - k_{m,1}T_2 - k_{m,2}T_1 \\
c_1 &= T_1R_{\text{tot}}(k_{m,2} - k_{m,1}) + k_{m,1}T_1T_2 + k_{m,2}T_1^2 + k_{m,1}k_{m,2}T_1 + k_{m,2}R_{\text{tot}}T_1 \\
d_1 &= -k_{m,2}R_{\text{tot}}T_1^2
\end{aligned}$$

and

$$\begin{aligned}
\Psi_2(x) &= a_2x^3 + b_2x^2 + c_2x + d_2 \\
a_2 &= k_{m,1} - k_{m,2} \\
b_2 &= -(k_{m,1} - k_{m,2})(R_{\text{tot}} + T_2 + k_{m,2}) - k_{m,2}T_1 - k_{m,1}T_2 \\
c_2 &= T_2R_{\text{tot}}(k_{m,1} - k_{m,2}) + k_{m,2}T_2T_1 + k_{m,1}T_2^2 + k_{m,2}k_{m,1}T_2 + k_{m,1}R_{\text{tot}}T_2 \\
d_2 &= -k_{m,1}R_{\text{tot}}T_2^2
\end{aligned}$$

For a biologically meaningful solution, we refer to the solution of $\Psi_1(RC_1) = 0$, $\Psi_2(RC_2) = 0$ where $(RC_1, RC_2) \in [0, \min\{T_1, R_{\text{tot}}\}] \times [0, \min\{T_2, R_{\text{tot}}\}]$. We will prove that $\Psi_1(RC_1) = 0$, $\Psi_2(RC_2) = 0$ has a unique biologically meaningful solution. To prove so, without loss of generality, we divide it into two cases; $k_{m,1} = k_{m,2}$, $k_{m,1} < k_{m,2}$.

1.1 Case 1: $k_{m,1} = k_{m,2}$

If $k_{m,1} = k_{m,2} = k_m$ then $a_1 = a_2 = 0$; hence $\Psi_1(x)$ and $\Psi_2(x)$ become quadratic equations. Therefore, solving $\Psi_1(x)$ by the quadratic formula, we obtain two solutions:

$$\begin{aligned}
z_1 &= \frac{T_1}{2(T_1+T_2)}\left(T_1 + T_2 + R_{\text{tot}} + k_m - \sqrt{(T_1 + T_2 + R_{\text{tot}} + k_m)^2 - 4R_{\text{tot}}(T_1+T_2)}\right) \\
z_2 &= \frac{T_1}{2(T_1+T_2)}\left(T_1 + T_2 + R_{\text{tot}} + k_m + \sqrt{(T_1 + T_2 + R_{\text{tot}} + k_m)^2 - 4R_{\text{tot}}(T_1+T_2)}\right)
\end{aligned}$$

Since $(T_1 + T_2 + R_{\text{tot}} + k_m)^2 - 4R_{\text{tot}}(T_1 + T_2) > (T_1 + T_2 + k_m - R_{\text{tot}})^2$,

$$\begin{aligned}
z_2 &= \frac{T_1}{2(T_1+T_2)}\left(T_1 + T_2 + R_{\text{tot}} + k_m + \sqrt{(T_1 + T_2 + R_{\text{tot}} + k_m)^2 - 4R_{\text{tot}}(T_1+T_2)}\right) && (1)\\
&> \frac{T_1}{2(T_1+T_2)}\left(T_1 + T_2 + R_{\text{tot}} + k_m + |T_1 + T_2 + k_m - R_{\text{tot}}|\right) && (2)\\
&\geq \frac{T_1}{2(T_1+T_2)}\left(T_1 + T_2 + R_{\text{tot}} + k_m + T_1 + T_2 + k_m - R_{\text{tot}}\right) && (3)\\
&= \frac{T_1}{2(T_1+T_2)}\left(2(T_1+T_2) + 2k_m\right) && (4)\\
&> \frac{T_1}{2(T_1+T_2)} \cdot 2(T_1+T_2) = T_1 && (5)
\end{aligned}$$

Hence z_2 is biologically meaningless. For z_1, $z_1 > 0$ and

$$z_1 = \frac{T_1}{2(T_1+T_2)}\left(T_1+T_2+R_{\text{tot}}+k_m-\sqrt{(T_1+T_2+R_{\text{tot}}+k_m)^2-4R_{\text{tot}}(T_1+T_2)}\right) \tag{6}$$

$$< \frac{T_1}{2(T_1+T_2)}\left(T_1+T_2+R_{\text{tot}}+k_m-|T_1+T_2+k_m-R_{\text{tot}}|\right) \tag{7}$$

$$\leq \frac{T_1}{2(T_1+T_2)}\left(T_1+T_2+R_{\text{tot}}+k_m-T_1-T_2-k_m+R_{\text{tot}}\right) \tag{8}$$

$$= \frac{T_1}{T_1+T_2}R_{\text{tot}} \leq R_{\text{tot}}, \tag{9}$$

thus $z_1 < R_{\text{tot}}$. Also

$$z_1 = \frac{T_1}{2(T_1+T_2)}\left(T_1+T_2+R_{\text{tot}}+k_m-\sqrt{(T_1+T_2+R_{\text{tot}}+k_m)^2-4R_{\text{tot}}(T_1+T_2)}\right) \tag{10}$$

$$< \frac{T_1}{2(T_1+T_2)}\left(T_1+T_2+R_{\text{tot}}+k_m-\sqrt{(T_1+T_2+R_{\text{tot}}+k_m)^2-4(R_{\text{tot}}+k_m)(T_1+T_2)}\right) \tag{11}$$

$$= \frac{T_1}{2(T_1+T_2)}\left(T_1+T_2+R_{\text{tot}}+k_m-|R_{\text{tot}}+k_m-T_1-T_2|\right) \tag{12}$$

$$\leq \frac{T_1}{2(T_1+T_2)}\left(T_1+T_2+R_{\text{tot}}+k_m-R_{\text{tot}}-k_m+T_1+T_2\right) = T_1 \tag{13}$$

and $z_1 < T_1$. Therefore $0 < z_1 < \min\{T_1, R_{\text{tot}}\}$ and z_1 is the biologically meaningful solution.

1.2 Case 2: $k_{m,1} < k_{m,2}$

We obtain the sign of each coefficient of $\Psi_1(x)$, $\Psi_2(x)$ as the following:

- $a_1 > 0$, $b_1 < 0$, $c_1 > 0$, $d_1 < 0$
- $a_2 < 0$, $d_2 < 0$

Lemma 1. *$\Psi_1(x)$ has three distinct real roots.*

Proof. Consider $\Psi_2(x)$ then

- $\lim_{x\to-\infty} \Psi_2(x) = \infty$
- $\Psi_2(0) = d_2 < 0$
- $\Psi_2(T_2) = k_{m,2}^2 T_2^2 > 0$
- $\lim_{x\to\infty} \Psi_2(x) = -\infty$

Therefore, by the Intermediate Value Theorem, $\Psi_2(x)$ has three distinct roots y_1, y_2, y_3 such that

- $y_1 \in (-\infty, 0)$
- $y_2 \in (0, T_2)$
- $y_3 \in (T_2, \infty)$

Since $\rho_2(y_i)$ is a root of $\Psi_1(x)$, $\Psi_1(x)$ has three distinct real roots $\rho_2(y_1)$, $\rho_2(y_2)$, $\rho_2(y_3)$. □

Lemma 2. *All roots of $\Psi_1(x)$ are positive.*

Proof. Let α, β, γ be distinct real roots of $\Psi_1(x)$. Without loss of generality, assume $\alpha < \beta < \gamma$. Then by Vieta's formula,

$$\alpha + \beta + \gamma = -\frac{b_1}{a_1} > 0 \tag{14}$$

$$\alpha\beta + \beta\gamma + \gamma\alpha = \frac{c_1}{a_1} > 0 \tag{15}$$

$$\alpha\beta\gamma = -\frac{d_1}{a_1} > 0 \tag{16}$$

By equation (16), $\alpha, \beta, \gamma > 0$ or $\gamma > 0$, $\alpha, \beta < 0$. Suppose $\gamma > 0$, $\alpha, \beta < 0$. Then by equation (14), $\gamma > -(\alpha + \beta)$ and

$$\beta\gamma < -\beta(\alpha + \beta), \ \gamma\alpha < -\alpha(\alpha + \beta)$$

since $\alpha, \beta < 0$. Substituting to equation (15),

$$\begin{aligned} 0 < \alpha\beta + \beta\gamma + \gamma\alpha &< \alpha\beta - (\alpha + \beta)^2 \\ &= -\alpha\beta - \alpha^2 - \beta^2 \end{aligned}$$

and $\alpha^2 + \beta^2 < -\alpha\beta < 0$, which is a contradiction. Therefore, $\alpha, \beta, \gamma > 0$. □

Lemma 3. *$\Psi_1(x)$ has at least one biologically meaningless solution.*

Proof. Since $y_1 < 0$ from Lemma 1,

$$\rho_2(y_1) = R_{\text{tot}} - y_1 - \frac{k_{m,2}y_1}{T_2 - y_1} > R_{\text{tot}}$$

and $\rho_2(y_1) \notin [0, \min\{T_1, R_{\text{tot}}\}]$. Hence $\rho_2(y_1)$ is a biologically meaningless root of $\Psi_1(x)$. □

Lemma 4. *The smallest root of $\Psi_1(x)$ is the unique biologically meaningful solution.*

Proof. Recall that $\Psi_1(x)$ satisfies the following properties:

- $\lim_{x \to -\infty} \Psi_1(x) = -\infty$
- $\Psi_1(0) = d_1 < 0$
- $\Psi_1(T_1) = k_{m,1}^2 T_1^2 > 0$
- $\Psi_1(R_{\text{tot}}) = (T_1 - R_{\text{tot}})(k_{m,1}k_{m,2}R_{\text{tot}} + k_{m,1}T_2R_{\text{tot}}) + k_{m,1}^2 R_{\text{tot}}^2$
- $\lim_{x \to \infty} \Psi_1(x) = \infty$

Since positivity of $\Psi_1(R_{\text{tot}})$ depends on T_1 and R_{tot}, we consider three cases: $T_1 = R_{\text{tot}}$, $T_1 < R_{\text{tot}}$ and $T_1 > R_{\text{tot}}$.

- **Case 1:** $T_1 = R_{\text{tot}}$

  Since $\Psi_1(0) < 0$ and $\Psi_1(T_1) > 0$ there exists one or three roots in $(0, T_1)$ by the Intermediate Value Theorem. However, by Lemma 3 there must exist at least one root in (R_{tot}, ∞); thus, only one root x_1 can be in $(0, T_1)$. Hence the smallest root x_1 is the unique biologically meaningful solution.

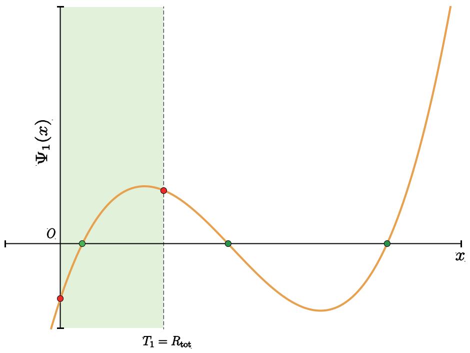

Figure 1: Position of roots of $\Psi_1(x)$ when $T_1 = R_{\text{tot}}$

- **Case 2:** $T_1 < R_{\text{tot}}$

  This implies that $\min\{T_1, R_{\text{tot}}\} = T_1$. Since $\Psi_1(0) < 0$ and $\Psi_1(T_1) > 0$ there exists one or three roots in $(0, T_1)$ by the Intermediate Value Theorem. However, by Lemma 3 there must exist at least one root in (R_{tot}, ∞); thus, only one root x_1 can be in $(0, T_1)$. Hence the smallest root x_1 is the unique biologically meaningful solution.

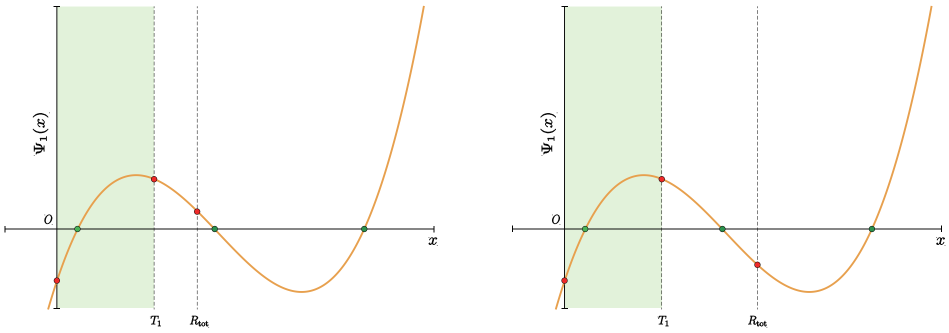

Figure 2: Position of roots of $\Psi_1(x)$ when $T_1 < R_{\text{tot}}$

- **Case 3:** $T_1 > R_{\text{tot}}$ This implies that $\min\{T_1, R_{\text{tot}}\} = R_{\text{tot}}$. Since $\Psi_1(0) < 0$ and $\Psi_1(R_{\text{tot}}) > 0$ there exists one or three roots in $(0, R_{\text{tot}})$ by the Intermediate Value Theorem. However, by Lemma 3 there must exist at least one root in (R_{tot}, ∞); thus, only one root x_1 can be in $(0, R_{\text{tot}})$. Hence the smallest root x_1 is the unique biologically meaningful solution.

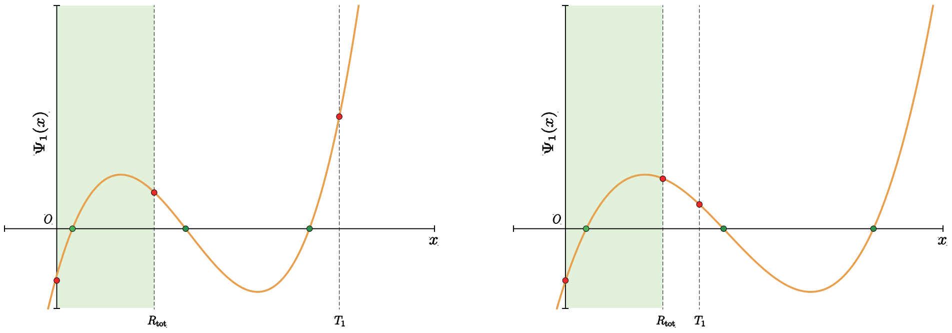

Figure 3: Position of roots of $\Psi_1(x)$ when $T_1 > R_{\text{tot}}$

Therefore for each case, there exists a unique biologically meaningful solution of $\Psi_1(x)$ and it is the smallest root among three. □

By the cubic formula, we obtain three roots of $\Psi_1(x)$ as the following:

$$z_1 = -\frac{1}{3a_1}(b_1 + P + M) \tag{17}$$

$$z_2 = -\frac{1}{3a_1}(b_1 + e^{2\pi/3i}P + e^{4\pi/3i}M) \tag{18}$$

$$z_3 = -\frac{1}{3a_1}(b_1 + e^{4\pi/3i}P + e^{2\pi/3i}M) \tag{19}$$

where Δ is the discriminant given as:

$$\Delta = a_1^4(z_1 - z_2)^2(z_1 - z_3)^2(z_2 - z_3)^2 \tag{20}$$

$$= b_1^2c_1^2 - 4a_1c_1^3 - 4b_1^3d_1 - 27a_1^2d_1^2 + 18a_1b_1c_1d_1 \tag{21}$$

and for $A = 2b_1^3 - 9a_1b_1c_1 + 27a_1^2d_1$, $B = \sqrt{-27a_1^2\Delta}$, P, M is given as:

$$P = \sqrt[3]{\frac{A+B}{2}} \tag{22}$$

$$M = \sqrt[3]{\frac{A-B}{2}} \tag{23}$$

As Ψ_1 has three distinct real roots by Lemma 1, $\Delta > 0$ and B is a pure imaginary number, therefore P and M are complex conjugates.

Theorem 1. *When $k_{m,1} < k_{m,2}$, then a competitive qTMDD system has a unique biologically meaningful solution $(RC_1, RC_2) = (x_1, \rho_1(x_1))$.*

Proof. By Lemma 4, it is sufficient to prove that z_1 is the smallest root among three. Let $R = \mathrm{Re}(P)$ and $J = \mathrm{Im}(P)$.

$$z_1 = -\frac{1}{3a_1}(b + 2R) \tag{24}$$

$$z_2 = -\frac{1}{3a_1}(b - R - \sqrt{3}J) \tag{25}$$

$$z_3 = -\frac{1}{3a_1}(b - R + \sqrt{3}J) \tag{26}$$

Let $(A + B)/2 = re^{i\theta}$ for $r > 0$ and $0 \leq \theta \leq 2\pi$. Moreover, since

$$\sin\theta = \mathrm{Im}\left(\frac{A + B}{2}\right) = \frac{1}{2}\sqrt{27a_1^2\Delta} \geq 0 \tag{27}$$

$0 \leq \theta \leq \pi$. Also $(A - B)/2 = re^{-i\theta}$ thus

$$P = R + iJ = \sqrt[3]{re^{i\theta}} = \sqrt[3]{r}e^{\theta/3i} \tag{28}$$

$$M = R - iJ = \sqrt[3]{re^{-i\theta}} = \sqrt[3]{r}e^{-\theta/3i} \tag{29}$$

Because $0 \leq \theta/3 \leq \pi/3$, $R = \sqrt[3]{r}\cos(\theta/3) \geq 0$ and $J = \sqrt[3]{r}\sin(\theta/3) \geq 0$, and

$$J/R = \tan(\theta/3) \leq \tan(\pi/3) = \sqrt{3}. \tag{30}$$

Then,

$$z_2 - z_1 = \frac{1}{3a_1}(3R + \sqrt{3}J) \geq 0. \tag{31}$$

Also,

$$z_3 - z_1 = \frac{1}{3a_1}(3R - \sqrt{3}J) = \frac{1}{\sqrt{3}a_1}(\sqrt{3}R - J) \geq 0. \tag{32}$$

Therefore $z_1 \leq z_2$ and $z_1 \leq z_3$ thus z_1 is the smallest root among three and is the unique biologically meaningful root. □

This proof can also be visualized as the following.

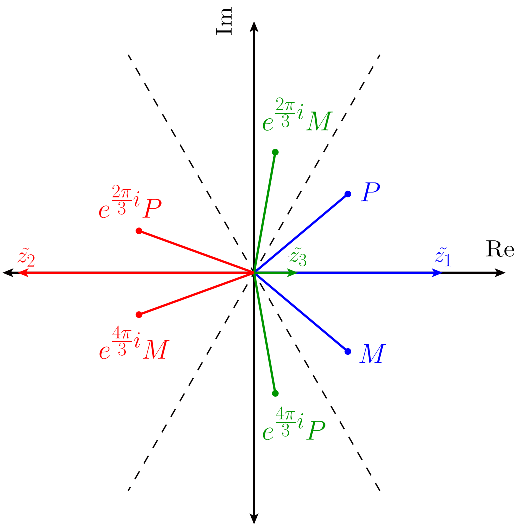

Figure 4: Geometry of P, M and $\tilde{z_1}, \tilde{z_2}, \tilde{z_3}$ on the complex plane

Let $\tilde{z_1} = P + M$, $\tilde{z_2} = e^{2\pi/3i}P + e^{4\pi/3i}M$, $\tilde{z_3} = e^{4\pi/3i}P + e^{2\pi/3i}M$ so that $z_j = -\frac{1}{3a_1}(b_1 + \tilde{z_j})$ for $j = 1, 2, 3$. Then, $\tilde{z_1} = 2\,\mathrm{Re}(P)$, $\tilde{z_2} = 2\,\mathrm{Re}(e^{2\pi/3i}P)$, $\tilde{z_3} = 2\,\mathrm{Re}(e^{4\pi/3i}P)$. Since $0 \leq \mathrm{Arg}(P) \leq \pi/3$, $2\pi/3 \leq \mathrm{Arg}(e^{2\pi/3i}P) \leq \pi$ and $4\pi/3 \leq \mathrm{Arg}(e^{4\pi/3i}P) \leq 5\pi/3$. Therefore,

$$\mathrm{Re}(P) \geq 1/2 \geq \mathrm{Re}(e^{2\pi/3i}P), \mathrm{Re}(e^{4\pi/3i}P) \tag{33}$$

and $\tilde{z_1} \geq \tilde{z_2}$, $\tilde{z_1} \geq \tilde{z_3}$. Because $a_1 > 0$, $z_1 \leq z_2$, $z_1 \leq z_3$ and z_1 is the smallest root among three.

2 Explicit Real Form of the Solution

From the proof of Theorem 1, we have shown that

$$z_1 = -\frac{1}{3a_1}(b_1 + 2\mathrm{Re}(P)) \tag{34}$$

Also since $\Delta > 0$,

$$P = \sqrt[3]{\frac{A+B}{2}} = \frac{1}{\sqrt[3]{2}}\sqrt[3]{A+B} = \frac{1}{\sqrt[3]{2}}\sqrt[3]{A + \sqrt{27a_1^2\Delta}i} \tag{35}$$

Let $\psi = A + i\sqrt{27a_1^2\Delta}$ then $\psi = \sqrt{A^2 + 27a_1^2\Delta}e^{i\theta}$ for $\theta = \mathrm{atan2}(\sqrt{27a_1^2\Delta}, A)$ using the $\mathrm{atan2}(y, x)$ convention. Therefore,

$$P = \frac{1}{\sqrt[3]{2}}\psi^{1/3} = \frac{1}{\sqrt[3]{2}}\left(A^2 + 27a_1^2\Delta\right)^{1/6} e^{i\theta/3} \tag{36}$$

and $\mathrm{Re}(P) = \frac{1}{\sqrt[3]{2}}\left(A^2 + 27a_1^2\Delta\right)^{1/6}\cos(\theta/3)$. Thus, we may obtain that

$$z_1 = -\frac{1}{3a_1}\left(b_1 + \sqrt[3]{4}\left(A^2 + 27a_1^2\Delta\right)^{1/6}\cos\left(\frac{1}{3}\mathrm{atan2}(\sqrt{27a_1^2\Delta}, A)\right)\right) \tag{37}$$

where every term is real.

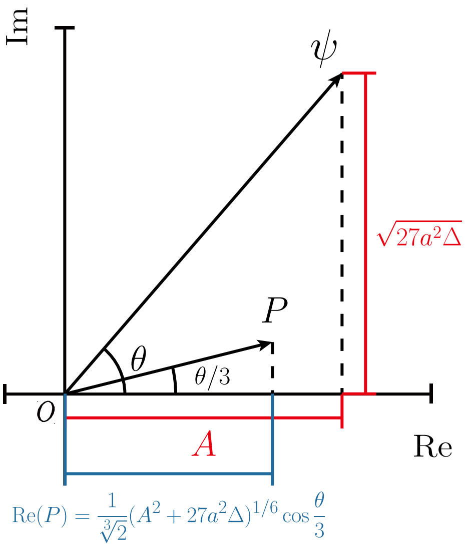

Figure 5: Geometry of ψ and P on the complex plane

The MATLAB implementation of this explicit real form of the solution can be found in the supplementary materials. When arctan is used instead of atan2 in the implementation, π must be added to $\arctan(\sqrt{27a_1^2\Delta} \cdot 1/A)$ when $A < 0$, and one must set the value to $\pi/2$ when $A = 0$.

During the implementation, when $\Delta \approx 0$, numerical round-off errors may lead to small negative values of Δ, which would prevent evaluation of its sqaure root. This can be resolved by using $\max\{\Delta, 0\}$ instead of Δ in the formula.

Also, when $|k_{m,2} - k_{m,1}|$ is below a numerical tolerance $\varepsilon \cdot \max\{k_{m,1}, k_{m,2}\}$ for small $\varepsilon > 0$, it is recommended to use the quadratic formula (from the case $k_{m,1} = k_{m,2}$) to avoid division by a nearly zero cubic coefficient.

3 Reduction to the Single-Drug Case

In the extreme case when $C_2 = 0$, we show that the competitive qTMDD reduces into single-drug qTMDD. Single-drug TMDD system is given as

$$\frac{\mathrm{d}}{\mathrm{d}t}C = -k_{\mathrm{el}}C - k_{\mathrm{on}}(R_{\mathrm{tot}} - RC)C + k_{\mathrm{off}}RC$$
$$\frac{\mathrm{d}}{\mathrm{d}t}RC = k_{\mathrm{on}}(R_{\mathrm{tot}} - RC)C - (k_{\mathrm{off}} + k_{\mathrm{int}})RC$$

and as in competitive qTMDD, by letting $T = C + RC$ and substituting $T - RC$ for C in $\mathrm{d}RC/\mathrm{d}t = 0$, we obtain the equation for the single-drug qTMDD:

$$RC^2 - (R_{\mathrm{tot}} + T + k_m)RC + TR_{\mathrm{tot}} = 0 \tag{38}$$

where $k_m = (k_{\mathrm{off}} + k_{\mathrm{int}})/k_{\mathrm{on}}$.

By the assumption $C_2 = RC_2 = 0$ (hence $T_2 = 0$), the coefficients for $\Psi_1(x)$ are given as

$$a_1 = k_{m,2} - k_{m,1} \tag{39}$$
$$b_1 = -(k_{m,2} - k_{m,1})(R_{\mathrm{tot}} + T_1 + k_{m,1}) - k_{m,2}T_1 \tag{40}$$
$$c_1 = T_1R_{\mathrm{tot}}(k_{m,2} - k_{m,1}) + k_{m,2}T_1^2 + k_{m,1}k_{m,2}T_1 + k_{m,2}R_{\mathrm{tot}}T_1 \tag{41}$$
$$d_1 = -k_{m,2}R_{\mathrm{tot}}T_1^2 \tag{42}$$

Let $U = k_{m,2} - k_{m,1}$, $V = R_{\mathrm{tot}} + T_1 + k_{m,1}$ then

$$\Psi_1(x) = Ux^3 + (-UV - k_{m,2}T_1)x^2 + (T_1R_{\mathrm{tot}}U + k_{m,2}T_1V)x - k_{m,2}R_{\mathrm{tot}}T_1^2 \tag{43}$$
$$= (Ux - k_{m,2}T_1)(x^2 - Vx + T_1R_{\mathrm{tot}}) \tag{44}$$

Assume $k_{m,1} \neq k_{m,2}$ i.e. $U \neq 0$. Then, $\Psi_1(x) = 0$ if and only if $x = \dfrac{k_{m,2}T_1}{U} = \dfrac{k_{m,2}T_1}{k_{m,2} - k_{m,1}}$ or

$$x^2 - Vx + T_1R_{\mathrm{tot}} = x^2 - (R_{\mathrm{tot}} + T_1 + k_{m,1})x + T_1R_{\mathrm{tot}} = 0$$

Suppose $x = \dfrac{k_{m,2}T_1}{k_{m,2} - k_{m,1}}$. If $k_{m,2} > k_{m,1}$ then $\dfrac{k_{m,2}}{k_{m,2} - k_{m,1}} > 1$, thus $x > T_1$ which is not a biologically meaningful solution. If $k_{m,2} < k_{m,1}$, $x < 0$, which is not biologically meaningful. Therefore, the only biologically meaningful solution is obtained from

$$x^2 - (R_{\mathrm{tot}} + T_1 + k_{m,1})x + T_1R_{\mathrm{tot}} = 0 \tag{45}$$

which is the qTMDD equation for the single-drug TMDD system for drug 1. When $k_{m,1} = k_{m,2}$ i.e. $U = 0$, $\Psi_1(x) = 0$ if and only if equation (44) is satisfied, hence the system also reduces to single-drug case.

4 Pseudocode for the Bisection Method

Algorithm 1 Bisection Method $(T_1, T_2, R_{\text{tot}}, k_{\text{m},1}, k_{\text{m},2}, n, \varepsilon)$

$l \leftarrow 0$
$r \leftarrow \min(T_1, R_{\text{tot}})$
$k \leftarrow 0$

while $k \leq n$ **do**
    $m \leftarrow (l + r)/2$

    if $\Psi_1(m) = 0$ or $l - r < \varepsilon$ **then**
        break
    end if

    if $\text{sgn}(\Psi_1(l)) = \text{sgn}(\Psi_1(m))$ **then**
        $l \leftarrow m$
    else
        $r \leftarrow m$
    end if
end while

return m
