# Supplementary material for: Exact Formula of the Total Quasi-Steady State Approximation in Competitive Target-Mediated Drug Disposition
Source: Comput Struct Biotechnol J. 2026 Oct 8;35(1):0223. doi: 10.34133/csbj.0223 (PMC13646460; doi:10.34133/csbj.0223)
Supplement: Supplementary file 1 [file csbj.0223.f1.zip › S10 Fig.pdf]

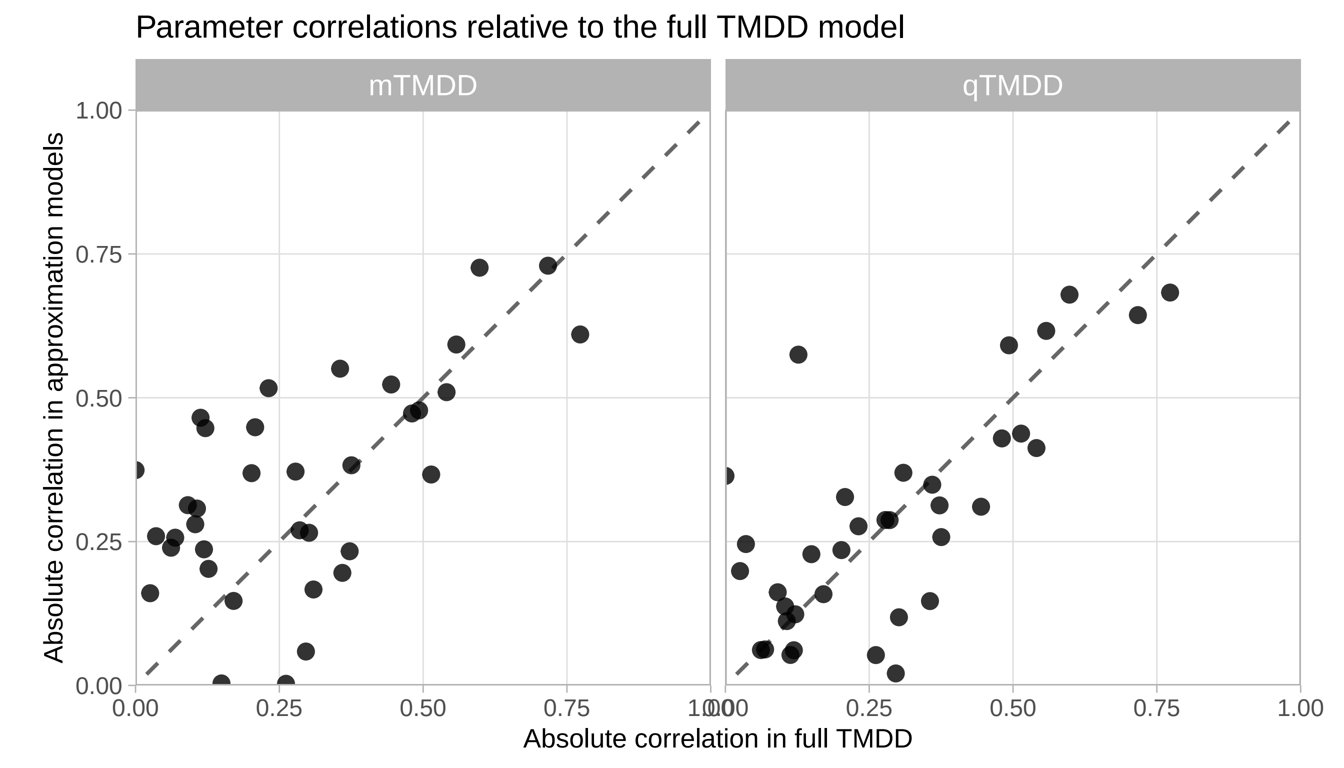

Parameter correlations relative to the full TMDD model
mTMDD
qTMDD
Absolute correlation in approximation models
Absolute correlation in full TMDD
0.00
0.25
0.50
0.75
1.00
