# Supplementary material for: Exact Formula of the Total Quasi-Steady State Approximation in Competitive Target-Mediated Drug Disposition
Source: Comput Struct Biotechnol J. 2026 Oct 8;35(1):0223. doi: 10.34133/csbj.0223 (PMC13646460; doi:10.34133/csbj.0223)
Supplement: Supplementary file 1 [file csbj.0223.f1.zip › S11 Fig.pdf]

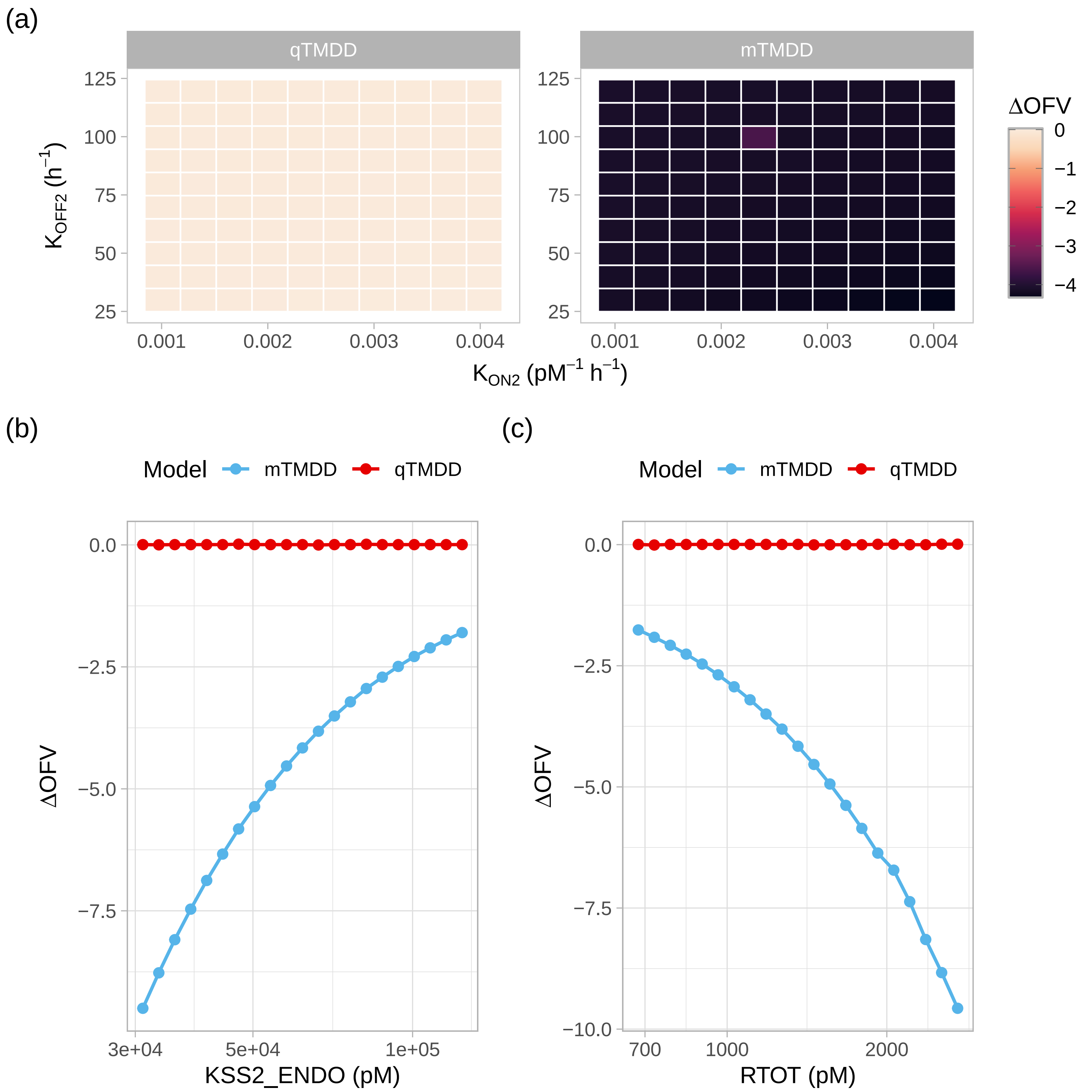

(a)
qTMDD
mTMDD
125
100
75
50
25
0.001
0.002
0.003
0.004
K_{OFF2} (h^{-1})
K_{ON2} (pM^{-1} h^{-1})
ΔOFV
0
−1
−2
−3
−4
(b)
(c)
Model
mTMDD
qTMDD
0.0
−2.5
−5.0
−7.5
−10.0
ΔOFV
3e+04
5e+04
1e+05
KSS2_ENDO (pM)
700
1000
2000
RTOT (pM)
