# Supplementary material for: Exact Formula of the Total Quasi-Steady State Approximation in Competitive Target-Mediated Drug Disposition
Source: Comput Struct Biotechnol J. 2026 Oct 8;35(1):0223. doi: 10.34133/csbj.0223 (PMC13646460; doi:10.34133/csbj.0223)
Supplement: Supplementary file 1 [file csbj.0223.f1.zip › S2 Table.pdf]

Table S2. Parameter estimate result and OFV value for Case 2 estimation results with dense simulated dataset

	Full TMDD		mTMDD		qTMDD	
OFV	7374.486		7374.14		7374.552	
Parameters (unit)						
CL (L/h)	11.4	(10.7%)	11.6	(15.4%)	11.4	(10.7%)
V_C (L)	29.3	(21.6%)	31.8	(29.1%)	29.2	(36.3%)
k_{a_im} (1/h)	0.00662	(3.5%)	0.00669	(4.0%)	0.00662	(5.5%)
k_{a_sc} (1/h)	0.00244	(6.7%)	0.00248	(11.4%)	0.00244	(9.2%)
k_{int} (1/h)	1.5	(1.9%)	1.35	(26.4%)	1.5	(38.7%)
V_D (L)	0.00154	(6.7%)	0.000508	(34.8%)	0.00115	(17.2%)
k_{on_endo} (1/(pM*h))	0.046	(9.7%)				
Inter-individual variability (variance)						
CL	0.182	(28.0%)	0.182	(31.3%)	0.182	(30.4%)
Vc	1.7	(20.8%)	1.74	(22.2%)	1.7	(22.6%)
Residual error (%)						
Proportional	35.8	(1.3%)	35.7	(1.3%)	35.8	(1.7%)

Values are point estimates; numbers in parentheses are the relative standard error (RSE, %). Values are point estimates; numbers in parentheses are the relative standard error (RSE, %). Refer to Table S1 for parameter definitions and details.
