# Supplementary material for: Exact Formula of the Total Quasi-Steady State Approximation in Competitive Target-Mediated Drug Disposition
Source: Comput Struct Biotechnol J. 2026 Oct 8;35(1):0223. doi: 10.34133/csbj.0223 (PMC13646460; doi:10.34133/csbj.0223)
Supplement: Supplementary file 1 [file csbj.0223.f1.zip › S2 Text.docx]

**S2 Text. The NONMEM code of the population pharmacokinetic model with each approximation method for competitive TMDD implemented.**

(a) The full TMDD model for Case 1 is as follows:

$PROBLEM    original

;; 1. Based on: run006_7

;; 2. Description: full TMDD model

;; x1. Author: hyeseon

$DATA      Table S3.csv IGNORE=@

$INPUT      ID TIME AMT DV CMT MDV EVID AGE BWKG HTCM BMI DVMG=DROP

$ABBREVIATED COMRES=2

$SUBROUTINE ADVAN13 TOL=9

$MODEL      NCOMP=5 COMP=(DEPOT,DEFDOSE) COMP=(CENTRAL)

            COMP=(CENTRAL_endo) COMP=(exo_cpx) COMP=(endo_cpx)

$PK

      CL    = THETA(1)* EXP(ETA(1))       ; Apparent clearance of free Anakinra from central compartment

      VC    = THETA(2)* EXP(ETA(2))       ; Apparent volume of distribution of free Anakinra in central compartment

      KA   = THETA(3)* EXP(ETA(3))        ; Absorption rate constant of Anakinra from injection site

      KEL  = CL/VC                        ; Elimination rate constant of free Anakinra from central compartment

      Kint = THETA(4)                     ; Degradation rate constant of complex Anakinra in central compartment

      Rtot  = THETA(5)                    ; Total amount (unbound- and bound-to Anakinra) of IL1R

      KON2  = THETA(9)

      KOFF2 = 0.75*KON2 - Kint

      KON_endo = 2.58 ; /nM*h

      KOFF_endo = 0.075 ; / h

      KSS_endo = (KOFF_endo+Kint)/KON_endo

      S2    = VC

      Kel_endo = THETA(8)

      ksyn = Kel_endo * 0.000147 + Kint * (Rtot/KSS_endo) * 0.000147

A_0(1) = 0

A_0(2) = 0.0000001

A_0(3) = 0.000147*VC

A_0(4) = 0.0000001

A_0(5) = 0.000147*(Rtot/KSS_endo)*VC

$DES

Cfree1 = A(2)/VC

Cfree2 = A(3)/VC

Ccpx1 = A(4)/VC

Ccpx2 = A(5)/VC

; note that ksyn = kel_endo*A(3) + kint_endo*A(5) for endogenous level to be same before and after administration

; ------Distribution1 interstital, vascular [4]

DADT(1) = - KA*A(1)                                                             ; drug, injection site

DADT(2) = KA*A(1) - KEL*A(2) - KON2*A(2)*(Rtot - Ccpx1 - Ccpx2) + KOFF2*A(4)    ; drug, cfree

DADT(3) = ksyn*VC - Kel_endo*A(3) - KON_endo*A(3)*(Rtot - Ccpx1 - Ccpx2) + KOFF_endo*A(5)    ; endogenous, cfree

DADT(4) = KON2*A(2)*(Rtot - Ccpx1 - Ccpx2) - (KOFF2 + Kint)*A(4)                            ; drug, ccpx

DADT(5) = KON_endo*A(3)*(Rtot - Ccpx1 - Ccpx2) - (KOFF_endo + Kint)*A(5)               ; endogenous, ccpx

$ERROR

      IPRED = A(2)/VC

      W     = SQRT(THETA(6)**2+THETA(7)**2*IPRED**2)

      IRES  = DV-IPRED

      IWRES = IPRED/W

      Y     = IPRED + W*EPS(1)

$THETA

(0, 9.69) ; CL    L/h

(0, 52.9) ; VC    L

(0, 0.448) ; KA    1/h

(0, 0.0717) ; Kint  1/h

(0, 1.54) FIX ; Rtot  nmol/L

(0) FIX ; Additive ERROR

(0, 0.116) ; Proportiona ERROR

(0, 0.046) FIX

(0, 3.07) FIX ; Kon   1/(nM*h)

$OMEGA  BLOCK(2)

 0.0108  ;         CL

 0.00101 0.113  ;         V2

$OMEGA 0.254 ;        KaA

$SIGMA 1 FIX

$COVARIANCE PRINT=E

$ESTIMATION NSIG=3 SIGL=9 PRINT=5 METHOD=1 MAX=9999 NOABORT INTER

$TABLE ID TIME DV  Cfree1 Cfree2 Ccpx1 Ccpx2 IPRED PRED IWRES CWRES ONEHEADER NOPRINT

FILE=sdtab001

$TABLE ID CL VC KA ONEHEADER NOPRINT FILE=patab001

$TABLE ID TIME IPRED IRES IWRES ONEHEADER NOPRINT FILE=table.txt

$TABLE ID ETA(1) ETA(2) ETA(3) ONEHEADER FIRSTONLY NOPRINT

NOAPPEND FILE=etatable.txt

;-----------------------

;TIME = h; DOSE = nmol; Cp = nmol/L;

(b) The mTMDD model for Case 1 is as follows:

$PROBLEM    mtmdd

;; 1. Based on: run021_1

;; 2. Description: mTMDD competitive model for Anakinra

;; x1. Author: hyeseon

$DATA      Table S3.csv IGNORE=@

$INPUT      ID TIME AMT DV CMT MDV EVID AGE BWKG HTCM BMI DVMG=DROP

$ABBREVIATED COMRES=2

$SUBROUTINE ADVAN13 TOL=9

$MODEL      NCOMP=3 COMP=(DEPOT,DEFDOSE) COMP=(CENTRAL) COMP=(endo)

$PK

      CL    = THETA(1)* EXP(ETA(1))       ; Apparent clearance of free Anakinra from central compartment

      VC    = THETA(2)* EXP(ETA(2))       ; Apparent volume of distribution of free Anakinra in central compartment

      KA   = THETA(3)* EXP(ETA(3))        ; Absorption rate constant of Anakinra from injection site

      KEL  = CL/VC                        ; Elimination rate constant of free Anakinra from central compartment

      Kint = THETA(4)                     ; Degradation rate constant of complex Anakinra in central compartment

      Rtot  = THETA(5)                    ; Total amount (unbound- and bound-to Anakinra) of IL1R

      KSS2 = 0.75

      Kel_endo = THETA(8)

      KON_endo = 2.58 ; /nM*h

      KOFF_endo = 0.075 ; / h

      KSS_endo = (KOFF_endo+Kint)/KON_endo

      S2    = VC

      ksyn = Kel_endo * 0.000147 + Kint * (Rtot/KSS_endo) * 0.000147

A_0(1) = 0

A_0(2) = 0.0000001

A_0(3) = 0.000147*(1+Rtot/KSS_endo)*VC

$DES

Cfree1 = A(2)/VC

Cfree2 = A(3)/VC

Ccpx1 = KSS_endo*Rtot*Cfree1/(KSS_endo*Cfree1 + KSS2*Cfree2 + KSS2*KSS_endo);

Ccpx2 = KSS2*Rtot*Cfree2/(KSS_endo*Cfree1 + KSS2*Cfree2 + KSS2*KSS_endo);

DADT(1) = - KA*A(1)

DADT(2) = KA*A(1)  - KEL*Cfree1*VC - Kint*Ccpx1*VC     ; drug

DADT(3) = ksyn*VC - kel_endo*Cfree2*VC - Kint*Ccpx2*VC               ; endogenous

$ERROR

Cf1 = A(2)/VC

IPRED = Cf1

W     = SQRT(THETA(6)**2+THETA(7)**2*IPRED**2)

IRES  = DV-IPRED

IWRES = IPRED/W

Y     = IPRED + W*EPS(1)

$THETA

(0, 9.97) ; CL    L/h

(0, 22.2) ; VC    L

(0, 0.182) ; KA    1/h

(0, 0.0494) ; Kint  1/h

(0, 1.54) FIX ; Rtot  nmol/L

(0) FIX ; Additive ERROR

(0, 0.116) ; Proportiona ERROR

(0, 0.046) FIX ;

$OMEGA  BLOCK(2)

 0.0108  ;         CL

 0.00623 0.206  ;         V2

$OMEGA 0.083 ;        KaA

$SIGMA 1 FIX

$COVARIANCE MATRIX=R PRINT=E

$ESTIMATION NSIG=3 SIGL=9 PRINT=5 METHOD=1 MAX=9999 NOABORT INTER

$TABLE ID TIME IPRED PRED Cfree1 Cfree2 Ccpx1 Ccpx2 IWRES CWRES

ONEHEADER NOPRINT FILE=sdtab002

$TABLE ID CL VC KA KSS2 ONEHEADER NOPRINT FILE=patab002

$TABLE ID TIME IPRED IRES IWRES ONEHEADER NOPRINT FILE=table.txt

$TABLE ID ETA(1) ETA(2) ETA(3) ONEHEADER FIRSTONLY NOPRINT

NOAPPEND FILE=etatable.txt

(c) The qTMDD model for Case 1 is as follows:

$PROBLEM    qTMDD

;; 1. Based on: run062

;; 2. Description: Explicit formula

;; x1. Author: hyeseon

$DATA      Table S3.csv IGNORE=@

$INPUT      ID TIME AMT DV CMT MDV EVID AGE BWKG HTCM BMI DVMG=DROP

$ABBREVIATED COMRES=2

$SUBROUTINE ADVAN13 TOL=9

$MODEL      NCOMP=3 COMP=(DEPOT,DEFDOSE) COMP=(CENTRAL) COMP=(endo)

$PK

      CL    = THETA(1)* EXP(ETA(1))       ; Apparent clearance of free Anakinra from central compartment

      VC    = THETA(2)* EXP(ETA(2))       ; Apparent volume of distribution of free Anakinra in central compartment

      KA   = THETA(3)* EXP(ETA(3))       ; Absorption rate constant of Anakinra from injection site

      KEL  = CL/VC                       ; Elimination rate constant of free Anakinra from central compartment

      Kint = THETA(4)                    ; Degradation rate constant of complex Anakinra in central compartment

      Rtot  = THETA(5)                    ; Total amount (unbound- and bound-to Anakinra) of IL1R

      KSS2 = 0.75

      Kel_endo = THETA(8)

      KON_endo = 2.58 ; /nM*h

      KOFF_endo = 0.075 ; / h

      KSS_endo = (KOFF_endo+Kint)/KON_endo

      S2    = VC

      ;Vcpx = THETA(10)

      ksyn = Kel_endo * 0.000147 + Kint * (Rtot/KSS_endo) * 0.000147

A_0(1) = 0

A_0(2) = 0.0000001

A_0(3) = 0.000147*(1+Rtot/KSS_endo)*VC

$DES

Ctot = A(2)/VC          ; exogenous

Ctot_endo = A(3)/VC         ; endogenous

a1 =  KSS2 - KSS_endo

b1 = -a1*(Rtot + Ctot_endo + KSS_endo) - KSS_endo*Ctot - KSS2*Ctot_endo

c1 = a1*Ctot_endo*Rtot + KSS_endo*Ctot*Ctot_endo + KSS2*(Ctot_endo**2) + KSS2*KSS_endo*Ctot_endo + KSS2*Rtot*Ctot_endo

d1 = -KSS2*Rtot*(Ctot_endo**2)

delta = (b1**2)*(c1**2) - 4*a1*(c1**3) - 4*(b1**3)*d1 - 27*(a1**2)*(d1**2) + 18*a1*b1*c1*d1

a2 = 2*(b1**3) -9*a1*b1*c1 + 27*(a1**2)*d1

y1 = sqrt(27*(a1**2)*delta)

theta_base = ATAN(y1/a2)

theta1 = theta_base

IF (a2.LT.0) theta1 = theta_base + 3.14159265358979

;IF (ABS(a2).LT.EPSS) theta1 = 1.5707963267949

Ccpx2 = (-1/(3*a1))*(b1 + 4**(1/3)*(a2**2 + 27*a1**2*delta)**(1/6)*COS(theta1/3)) ; endogenous

Cfree2 = Ctot_endo - Ccpx2                                                            ; endogenous

Ccpx1 = KSS_endo*Ccpx2*Ctot/((KSS_endo - KSS2)*Ccpx2 + KSS2*Ctot_endo)                ; exogenous

Cfree1 = Ctot - Ccpx1                                                             ; exogenous

DADT(1) = - KA*A(1)

DADT(2) = KA*A(1) - KEL*Cfree1*VC - Kint*Ccpx1*VC                 ; drug

DADT(3) = ksyn*VC - Kel_endo*Cfree2*VC - Kint*Ccpx2*VC               ; endogenous

$ERROR

Ct  = A(2)/VC

Ct2 = A(3)/VC

a11 =  KSS2 - KSS_endo

b11 = -a11*(Rtot+Ct2+KSS_endo) - KSS_endo*Ct - KSS2*Ct2

c11 = a11*Ct2*Rtot + KSS_endo*Ct*Ct2 + KSS2*(Ct2**2) + KSS2*KSS_endo*Ct2 + KSS2*Rtot*Ct2

d11 = -KSS2*Rtot*(Ct2**2)

delta1 = (b11**2)*(c11**2) - 4*a11*(c11**3) - 4*(b11**3)*d11 - 27*(a11**2)*(d11**2) + 18*a11*b11*c11*d11

a21 = 2*(b11**3) -9*a11*b11*c11 + 27*(a11**2)*d11

y2 = sqrt(27*(a11**2)*delta1)

thet_base2 = ATAN(y2/a21)

thet2 = thet_base2

IF (a21.LT.0) thet2 = thet_base2 + 3.14159265358979

Cp2 = (-1/(3*a11))*(b11 + 4**(1/3)*(a21**2 + 27*a11**2*delta1)**(1/6)*COS(thet2/3)) ; endogenous

Cf2 = Ct2 - Cp2                                                                     ; endogenous

Cp1 = KSS_endo*Cp2*Ct/((KSS_endo - KSS2)*Cp2 + KSS2*Ct2)                            ; exogenous

Cf1 = Ct - Cp1                                                                      ; exogenous

IPRED = Cf1

W     = SQRT(THETA(6)**2 + THETA(7)**2 * IPRED**2)

IRES  = DV-IPRED

IWRES = IPRED/W

Y     = IPRED + W*EPS(1)

$THETA

(0, 9.69) ; CL    L/h

(0, 52.9) ; VC    L

(0, 0.448) ; KA    1/h

(0, 0.0735) ; Kint  1/h

(0, 1.54) FIX ; Rtot  nmol/L

(0) FIX ; Additive ERROR

(0, 0.116) ; Proportiona ERROR

(0, 0.046) FIX ; kel_endo

$OMEGA BLOCK(2)

 0.0108  ;         CL

 0.000723 0.113  ;         V2

$OMEGA

 0.256 ;        KaA

$SIGMA 1 FIX

$COVARIANCE MATRIX=R PRINT=E

$ESTIMATION NSIG=3 SIGL=9 PRINT=5 METHOD=1 MAX=9999 NOABORT INTER

$TABLE ID TIME DV Cfree1 Cfree2 Ccpx1 Ccpx2 IPRED PRED IWRES CWRES ONEHEADER NOPRINT FILE=sdtab003

$TABLE ID CL VC KA KSS2 ONEHEADER NOPRINT FILE=patab003

$TABLE ID TIME IPRED IRES IWRES ONEHEADER NOPRINT FILE=table.txt

$TABLE ID ETA(1) ETA(2) ETA(3) ONEHEADER FIRSTONLY NOPRINT

NOAPPEND FILE=etatable.txt

(d) The full TMDD model for Case 2 is as follows:

$PROBLEM    full TMDD

;; 1. Based on: run016_retry3

;; 2. Description: vpc

;; x1. Author: hyeseon

$INPUT      ID TIME DROP DROP DROP AMT DROP DROP DROP MDV COHORT CMT

            AGE SEX HTCM WTKG BMI DOSE B=DROP DV

$DATA      Table S4.csv IGNORE=#

$SUBROUTINE ADVAN13 TOL=9

$MODEL      COMP(DISTRIBUTION1) ; Injection site

            COMP(CENTRAL) COMP(endo) COMP(fcrn) COMP(CENTRAL_cpx)

            COMP(endo_cpx)

$PK

F1 = 2

BASE = 3.04

;--- Central compartment [3]

CL = THETA(1)*EXP(ETA(1))  ; Cl central

VC = THETA(2)*EXP(ETA(2))  ; Vd central

KEL = CL/VC

;------ TMDD in central [3]

Kint = THETA(4)            ; internalization of IL7R-drug complex (ref 0.642)

KOFF2 = 59.76              ; (1/h)

KON2 = 20.196/10000        ; (pM^-1 h*-1)

KSS2 = (KOFF2 + Kint)/KON2 ;(29590 pM = 29.590 nM)

Rtot = THETA(5)

KAIM = THETA(6)*EXP(ETA(3))

KASC = THETA(7)*EXP(ETA(4))

KA=KASC                    ; default VI (SC)

IF(COHORT.EQ.3) KA=KAIM    ; VI when cohort==3 (IM cohort)

KON_endo  = THETA(10)/10000

KOFF_endo = 62000 * KON_endo - Kint

Kel_endo  = Kint

Kint_endo = Kint

KSS2_endo = 62000 ; pM

;Ksyn = Kel_endo*BASE*(1+Rtot/KSS2_endo)

;-----FCRN recycling [5]

KOFF1 = 12.132             ; (1/h) dissociation

KON1 = 4.068/10000         ; (pM*-1h^-1) binding

KD = 29823                 ; (pmol/L)

Krec = THETA(8)            ; (1/h) recycle rate of drug-FcRn complex to central comp

FcRn = 91950000            ; Total amount of FcRn (pmol/L)

KSS1 = KD + Krec/KON1      ; (pmol/L) ; KSS1=(KOFF1 + Krec)

FR = 0.715                 ; recycling fraction of FcRn bound drug (ref)

VD = THETA(9)

Kup = 0.00952

A_0(2) = 0.000001

A_0(3) = BASE*VC

A_0(4) = 0.000001

A_0(5) = BASE*(Rtot/KSS2_endo)*VC

$DES

;-----FcRn mediated recycling

Atot = A(6)/VD             ; (pmol/L)

DF = Atot - FcRn - KSS1    ; (pmol/L)

Afree = 0.5 * (DF + SQRT(DF**2 + 4*KSS1*Atot)) ; AMT of active free drug (pmol/L) in distribution space derived from total amount indistribution space

                                               ; derived from total amount in distribution space

Acpx = FcRn * Afree/(KSS1 + Afree)

Cfree1 = A(2)/VC

Cfree2 = A(3)/VC

Ccpx1  = A(4)/VC

Ccpx2  = A(5)/VC

;ksyn = kel_endo*A(3) + Kint_endo*A(5)

; ------Distribution1 interstital, vascular [4]

DADT(1) = - KA*A(1)

DADT(2) = KA*A(1)  - KEL*A(2) - KON2*A(2)*(Rtot - Ccpx1 - Ccpx2) + KOFF2*A(4) - Kup*Cfree1*VC + Krec*Acpx*VD   ; drug, cfree

DADT(3) = - KON_endo*A(3)*(Rtot - Ccpx1 - Ccpx2) + KOFF_endo*A(5) + Kint*A(5)     ; endogenous, cfree

DADT(4) = KON2*A(2)*(Rtot - Ccpx1 - Ccpx2) - (KOFF2 + Kint)*A(4)                            ; drug, ccpx

DADT(5) = KON_endo*A(3)*(Rtot - Ccpx1 - Ccpx2) - (KOFF_endo + Kint)*A(5)               ; endogenous, ccpx

DADT(6) = Kup*Cfree1*VC - Krec*Acpx*VD

$ERROR

Cf1 = A(2)/VC

Cf2 = A(3)/VC

IPRED = Cf1+Cf2

W = SQRT(THETA(3)**2 * IPRED**2)

Y = IPRED + W*EPS(1)

IRES = IPRED - DV

IWRES = IRES/W

$THETA

(0, 13,100) ; 1.CL L/h

(0, 27.3) ; 2.VC L

(0, 0.357) ; 8.Prop

(0, 1.17) ; 4.Kint 1/h (ref)

(0, 1360) FIX ; 5.rtot

(0, 0.00774) ; 6.kaim

(0, 0.00239) ; 7.kasc

(0, 0.00107) FIX ; 8.krec

(0, 0.00295) ; 9.vd

(0, 0.0766,400) ;

$OMEGA 0.176

 1.68

 0 FIX

 0 FIX

$SIGMA 1 FIX

;$EST METHOD=SAEM AUTO=1 NITER=500

;$EST METHOD=IMP EONLY=1 NITER=20 ISAMPLE=200 MAPITER=0 NOABORT NSIG=3 SIGL=9

$COVARIANCE MATRIX=S PRINT=E

$ESTIMATION MAXEVAL=9999 PRINT=5 METHOD=1 INTER NOABORT NSIG=3 SIGL=9

$TABLE ID TIME CL VC KA AMT NOPRINT FILE=patab001

$TABLE ID TIME DV IPRED IWRES PRED CWRES Cfree1 Cfree2 Ccpx1 Ccpx2 NOPRINT

ONEHEADER FILE=sdtab001

(e) The mTMDD model for Case 2 is as follows:

$PROBLEM    mTMDD

;; 1. Based on: run093

;; 2. Description:

;; x1. Author: hyeseon

$INPUT      ID TIME DROP DROP DROP AMT DROP DROP DROP MDV COHORT CMT

            AGE SEX HTCM WTKG BMI DOSE B=DROP DV

$DATA      Table S4.csv IGNORE=#

$SUBROUTINE ADVAN13 TOL=9

$MODEL      COMP(DISTRIBUTION1) ; Injection site

            COMP(CENTRAL) COMP(endo) COMP(FcRn)

$PK

F1 = 2

BASE = 3.04

;--- Central compartment [3]

CL = THETA(1)*EXP(ETA(1))  ; Cl central

VC = THETA(2)*EXP(ETA(2))  ; Vd central

KEL = CL/VC

;---Endosomal space (Injection site & Lymph) [5]

;------ TMDD in central [3]

Kint = THETA(4)            ; internalization of IL7R-drug complex (ref 0.642)

KOFF2 = 59.76              ; (1/h)

KON2 = 20.196/10000        ; (pM^-1 h*-1)

KSS2 = (KOFF2 + Kint)/KON2 ;(29590 pM = 29.590 nM)

Rtot = THETA(5)

KAIM = THETA(6)*EXP(ETA(3))

KASC = THETA(7)*EXP(ETA(4))

KA=KASC                    ; default VI (SC)

IF(COHORT.EQ.3) KA=KAIM    ; VI when cohort==3 (IM cohort)

Kint_endo = Kint

Kel_endo  = Kint

KSS_endo = 62000 ; pM

;Ksyn = Kel_endo*BASE*(1+Rtot/KSS_endo)

A_0(2) = 0.000001

A_0(3) = BASE*(1 + Rtot/KSS_endo)*VC

;-----FCRN recycling [5]

KOFF1 = 12.132             ; (1/h) dissociation

KON1 = 4.068/10000         ; (pM*-1h^-1) binding

KD = 29823                 ; (pmol/L)

Krec = THETA(8)            ; (1/h) recycle rate of drug-FcRn complex to central comp

FcRn = 91950000            ; Total amount of FcRn (pmol/L)

KSS1 = KD + Krec/KON1      ; (pmol/L) ; KSS1=(KOFF1 + Krec)

FR = 0.715                 ; recycling fraction of FcRn bound drug (ref)

VD = THETA(9)

Kup = 0.00952

$DES

;-----FcRn mediated recycling

Atot = A(4)/VD             ; (pmol/L)

DF = Atot - FcRn - KSS1    ; (pmol/L)

Afree = 0.5 * (DF + SQRT(DF**2 + 4*KSS1*Atot)) ; AMT of active free drug (pmol/L) in distribution space derived from total amount indistribution space

                                               ; derived from total amount in distribution space

Acpx = FcRn * Afree/(KSS1 + Afree)

;-----TMDD with therapeutic receptor

Cfree1 = A(2)/VC

Cfree2 = A(3)/VC

Ccpx1 = KSS_endo*Rtot*Cfree1/(KSS_endo*Cfree1 + KSS2*Cfree2 + KSS2*KSS_endo);

Ccpx2 = KSS2*Rtot*Cfree2/(KSS_endo*Cfree1 + KSS2*Cfree2 + KSS2*KSS_endo);

DADT(1) = - KA*A(1)

DADT(2) = KA*A(1) - KEL*Cfree1*VC - Kint*Ccpx1*VC - Kup*Cfree1*VC + Krec*Acpx*VD   ; drug

DADT(3) = 0             ; endogenous

DADT(4) = Kup*Cfree1*VC - Krec*Acpx*VD

$ERROR

Cf1 = A(2)/VC

Cf2 = A(3)/VC

IPRED = Cf1 + Cf2

W = SQRT(THETA(3)**2 * IPRED**2)

Y = IPRED + W*EPS(1)

IRES = IPRED - DV

IWRES = IRES/W

$THETA

(0, 15.6) ; 1.CL L/h

(0, 36) ; 2.VC L

(0, 0.361) ; 8.Prop

(0, 0.455) ; 4.Kint 1/h (ref)

(0, 1360) FIX ; 5.rtot

(0, 0.0108) ; 6.kaim

(0, 0.00255) ; 7.kasc

(0, 0.00107) FIX ; 8.krec

(0, 0.00061) ; 9.vd

$OMEGA 0.192

 1.89

 0 FIX

 0 FIX

$SIGMA 1 FIX

;$EST METHOD=SAEM AUTO=1 NITER=500

;$EST METHOD=IMP EONLY=1 NITER=20 ISAMPLE=200 MAPITER=0 NOABORT NSIG=3 SIGL=9

$COVARIANCE MATRIX=S PRINT=E

$ESTIMATION MAXEVAL=9999 PRINT=5 METHOD=1 INTER NOABORT NSIG=3 SIGL=9

$TABLE ID TIME CL VC KA AMT NOPRINT FILE=patab002

$TABLE ID TIME DV IPRED IWRES PRED CWRES Cfree1 Cfree2 Ccpx1 Ccpx2 NOPRINT

ONEHEADER FILE=sdtab002

(f) The qTMDD model for Case 2 is as follows:

$PROBLEM    qTMDD

;; 1. Based on: run017s

;; 2. Description: qTMDD   vpc

;; x1. Author: hyeseon

$INPUT      ID TIME DROP DROP DROP AMT DROP DROP DROP MDV COHORT CMT

            AGE SEX HTCM WTKG BMI DOSE B=DROP DV

$DATA      Table S4.csv IGNORE=#

$SUBROUTINE ADVAN13 TOL=9

$MODEL      COMP(DISTRIBUTION1) ; Injection site

            COMP(CENTRAL) COMP(endo) COMP(FcRn)

$PK

F1 = 2

BASE = 3.04

;--- Central compartment [3]

CL = THETA(1)*EXP(ETA(1))  ; Cl central

VC = THETA(2)*EXP(ETA(2))  ; Vd central

KEL = CL/VC

;---Endosomal space (Injection site & Lymph) [5]

;------ TMDD in central [3]

Kint = THETA(4)            ; internalization of IL7R-drug complex (ref 0.642)

KOFF2 = 59.76              ; (1/h)

KON2 = 20.196/10000        ; (pM^-1 h*-1)

KSS2 = (KOFF2 + Kint)/KON2 ;(29590 pM = 29.590 nM)

Rtot = THETA(5)

KAIM = THETA(6)

KASC = THETA(7)

KA=KASC                    ; default VI (SC)

IF(COHORT.EQ.3) KA=KAIM    ; VI when cohort==3 (IM cohort)

Kint_endo = Kint

Kel_endo  = Kint

KSS2_endo = 62000 ; pM

Ksyn = Kel_endo*BASE*(1+Rtot/KSS2_endo)

;-----FCRN recycling [5]

KOFF1 = 12.132             ; (1/h) dissociation

KON1 = 4.068/10000         ; (pM*-1h^-1) binding

KD = 29823                 ; (pmol/L)

Krec = THETA(8)            ; (1/h) recycle rate of drug-FcRn complex to central comp

FcRn = 91950000            ; Total amount of FcRn (pmol/L)

KSS1 = KD + Krec/KON1      ; (pmol/L) ; KSS1=(KOFF1 + Krec)

FR = 0.715                 ; recycling fraction of FcRn bound drug (ref)

VD = THETA(9)

Kup = 0.00952

A_0(2) = 0.000001

A_0(3) = BASE*(1 + Rtot/KSS2_endo)*VC

$DES

;-----FcRn mediated recycling

Atot = A(4)/VD             ; (pmol/L)

DF = Atot - FcRn - KSS1    ; (pmol/L)

Afree = 0.5 * (DF + SQRT(DF**2 + 4*KSS1*Atot)) ; AMT of active free drug (pmol/L) in distribution space derived from total amount indistribution space

                                               ; derived from total amount in distribution space

Acpx = FcRn * Afree/(KSS1 + Afree)

Ctot = A(2)/VC          ; exogenous

Ctot2 = A(3)/VC         ; endogenous compound

a1 = KSS2_endo - KSS2

b1 = -a1*(Rtot+Ctot+KSS2) - KSS2*Ctot2 - KSS2_endo*Ctot

c1 = a1*Ctot*Rtot + KSS2*Ctot*Ctot2 + KSS2_endo*(Ctot**2) + KSS2*KSS2_endo*Ctot + KSS2_endo*Rtot*Ctot

d1 = -KSS2_endo*Rtot*(Ctot**2)

delta = (b1**2)*(c1**2) - 4*a1*(c1**3) - 4*(b1**3)*d1 - 27*(a1**2)*(d1**2) + 18*a1*b1*c1*d1

a2 = 2*(b1**3) -9*a1*b1*c1 + 27*(a1**2)*d1

y1 = sqrt(27*(a1**2)*delta)

theta_base = ATAN(y1/a2)

theta1 = theta_base

IF (a2.LT.0) theta1 = theta_base + 3.14159265358979

; exogenous drug

Ccpx1 = (-1/(3*a1))*(b1 + 4**(1/3)*(a2**2 + 27*a1**2*delta)**(1/6)*COS(theta1/3))

Cfree1 = Ctot - Ccpx1

; endogenous compound

Ccpx2 = KSS2*Ccpx1*Ctot2/((KSS2 - KSS2_endo)*Ccpx1 + KSS2_endo*Ctot)

Cfree2 = Ctot2 - Ccpx2

DADT(1) = - KA*A(1)

DADT(2) = KA*A(1) - KEL*Cfree1*VC - Kint*Ccpx1*VC - Kup*Cfree1*VC + Krec*Acpx*VD    ; drug

DADT(3) = 0              ; endogenous

DADT(4) = Kup*Cfree1*VC - Krec*Acpx*VD

$ERROR

Ct  = A(2)/VC ; (pmol/L)

Ct2 = A(3)/VC

a11 = KSS2_endo - KSS2

b11 = -a11*(Rtot + Ct + KSS2) - KSS2*Ct2 - KSS2_endo*Ct

c11 = a11*Ct*Rtot + KSS2*Ct*Ct2 + KSS2_endo*(Ct**2) + KSS2*KSS2_endo*Ct + KSS2_endo*Rtot*Ct

d11 = -KSS2_endo*Rtot*(Ct**2)

delta1 = (b11**2)*(c11**2) - 4*a11*(c11**3) - 4*(b11**3)*d11 - 27*(a11**2)*(d11**2) + 18*a11*b11*c11*d11

a21 = 2*(b11**3) -9*a11*b11*c11 + 27*(a11**2)*d11

y2 = SQRT(27*(a11**2)*delta1)

thet_base2 = ATAN(y2/a21)

thet2 = thet_base2

IF (a21.LT.0) thet2 = thet_base2 + 3.14159265358979

; exogenous

Cp1 = (-1/(3*a11))*(b11 + 4**(1/3)*(a21**2 + 27*a11**2*delta1)**(1/6)*COS(thet2/3))

Cf1 = Ct - Cp1

; endogenous

Cp2 = KSS2*Cp1*Ct2/((KSS2 - KSS2_endo)*Cp1 + KSS2_endo*Ct)

Cf2 = Ct2 - Cp2

IPRED = Cf1+Cf2

W = SQRT(THETA(3)**2 * IPRED**2)

Y = IPRED + W*EPS(1)

IRES = IPRED - DV

IWRES = IRES/W

$THETA

(0, 13) ; 1.CL L/h

(0, 27.4) ; 2.VC L

(0, 0.357) ; 8.Prop

(0, 1.17) ; 4.Kint 1/h (ref)

(0, 1360) FIX ; 5.rtot

(0, 0.00774) ; 6.kaim

(0, 0.00239) ; 7.kasc

(0, 0.00107) FIX ; 8.krec

(0, 0.00034) ; 9.vd

$OMEGA

 0.176

 1.68

$SIGMA 1 FIX

;$EST METHOD=SAEM AUTO=1 NITER=500

;$EST METHOD=IMP EONLY=1 NITER=20 ISAMPLE=200 MAPITER=0 NOABORT NSIG=3 SIGL=9

$COVARIANCE MATRIX=S PRINT=E

$ESTIMATION MAXEVAL=9999 PRINT=5 METHOD=1 INTER NOABORT NSIG=3 SIGL=9

$TABLE ID TIME CL VC KA AMT NOPRINT FILE=patab003

$TABLE ID TIME DV IPRED IWRES PRED CWRES Cfree1 Cfree2 Ccpx1 Ccpx2 NOPRINT

ONEHEADER FILE=sdtab003

W     = SQRT(THETA(7)**2 + THETA(8)**2 * IPRED**2)

IRES  = DV-IPRED

IWRES = IPRED/W

Y     = IPRED + W*EPS(1)

$THETA

(0, 9.69) ; CL    L/h

(0, 52.9) ; VC    L

(0, 0.448) ; KA    1/h

(0, 0.0735) ; Kint  1/h

(0, 1.54) FIX ; Rtot  nmol/L

(0, 0.046) FIX ; kel_endo

(0) FIX ; Additive ERROR

(0, 0.116) ; Proportiona ERROR

$OMEGA BLOCK(2)

 0.0108  ;         CL

 0.000723 0.113  ;         V2

$OMEGA

 0.256 ;        KaA

$SIGMA 1 FIX

$COVARIANCE MATRIX=R PRINT=E

$ESTIMATION NSIG=3 SIGL=9 PRINT=5 METHOD=1 MAX=9999 NOABORT INTER

$TABLE ID TIME DV Cfree1 Cfree2 Ccpx1 Ccpx2 IPRED PRED IWRES CWRES ONEHEADER NOPRINT FILE=sdtab006

$TABLE ID CL VC KA KSS2 ONEHEADER NOPRINT FILE=patab006

$TABLE ID TIME IPRED IRES IWRES ONEHEADER NOPRINT FILE=table.txt

$TABLE ID ETA(1) ETA(2) ETA(3) ONEHEADER FIRSTONLY NOPRINT

NOAPPEND FILE=etatable.txt
