# Supplementary material for: Exact Formula of the Total Quasi-Steady State Approximation in Competitive Target-Mediated Drug Disposition
Source: Comput Struct Biotechnol J. 2026 Oct 8;35(1):0223. doi: 10.34133/csbj.0223 (PMC13646460; doi:10.34133/csbj.0223)
Supplement: Supplementary file 1 [file csbj.0223.f1.zip › S3 Fig.pdf]

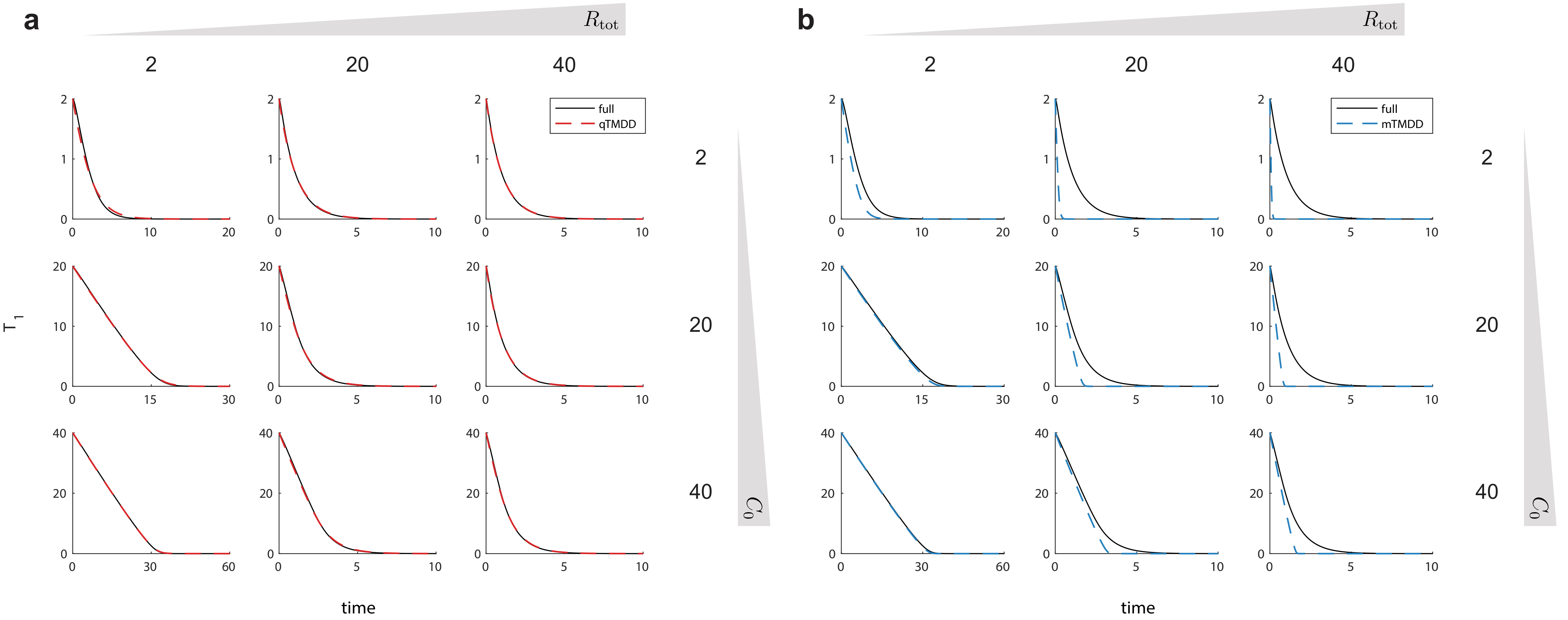

a
$R_{tot}$
2
20
40
full
qTMDD
$T_1$
time
$C_0$
b
$R_{tot}$
2
20
40
full
mTMDD
time
$C_0$
