# Supplementary material for: Exact Formula of the Total Quasi-Steady State Approximation in Competitive Target-Mediated Drug Disposition
Source: Comput Struct Biotechnol J. 2026 Oct 8;35(1):0223. doi: 10.34133/csbj.0223 (PMC13646460; doi:10.34133/csbj.0223)
Supplement: Supplementary file 1 [file csbj.0223.f1.zip › S4 Fig.pdf]

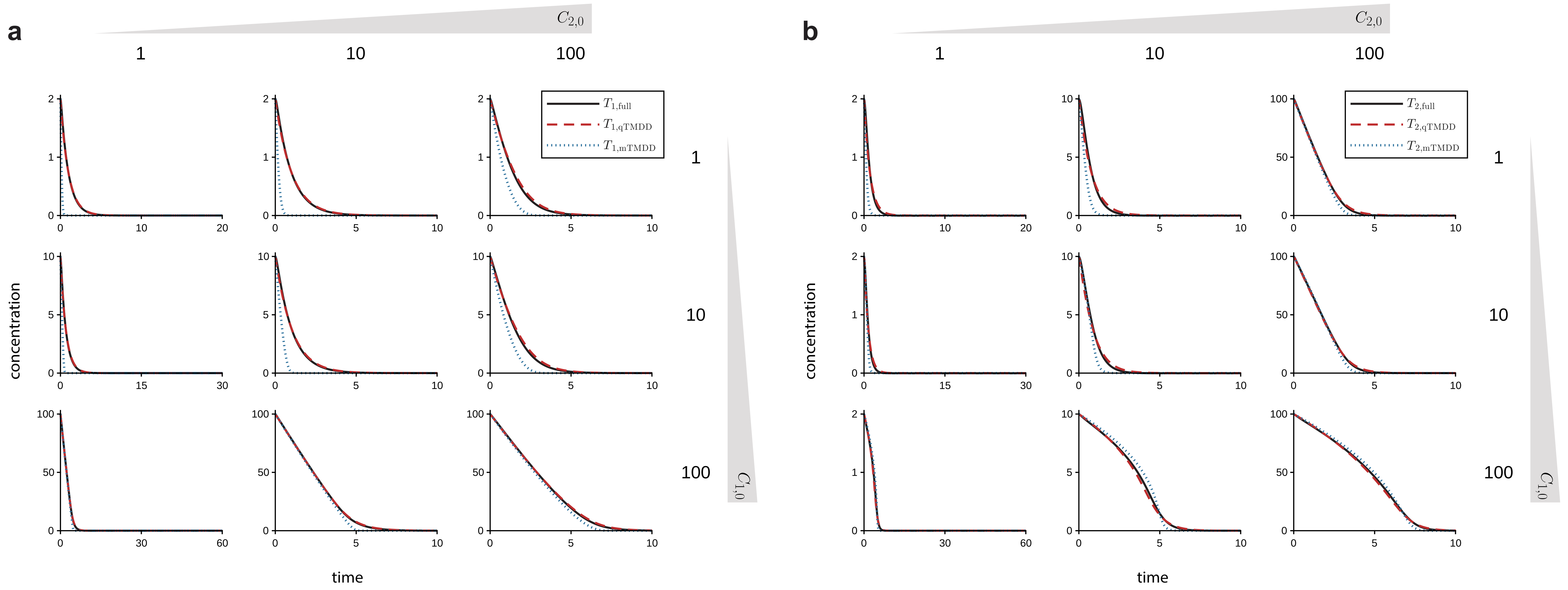

a
$C_{2,0}$
1
10
100
$T_{1,\mathrm{full}}$
$T_{1,\mathrm{qTMDD}}$
$T_{1,\mathrm{mTMDD}}$
1
10
100
$C_{1,0}$
concentration
time
b
$C_{2,0}$
1
10
100
$T_{2,\mathrm{full}}$
$T_{2,\mathrm{qTMDD}}$
$T_{2,\mathrm{mTMDD}}$
1
10
100
$C_{1,0}$
concentration
time
