# Supplementary material for: Exact Formula of the Total Quasi-Steady State Approximation in Competitive Target-Mediated Drug Disposition
Source: Comput Struct Biotechnol J. 2026 Oct 8;35(1):0223. doi: 10.34133/csbj.0223 (PMC13646460; doi:10.34133/csbj.0223)
Supplement: Supplementary file 1 [file csbj.0223.f1.zip › S5 Fig.pdf]

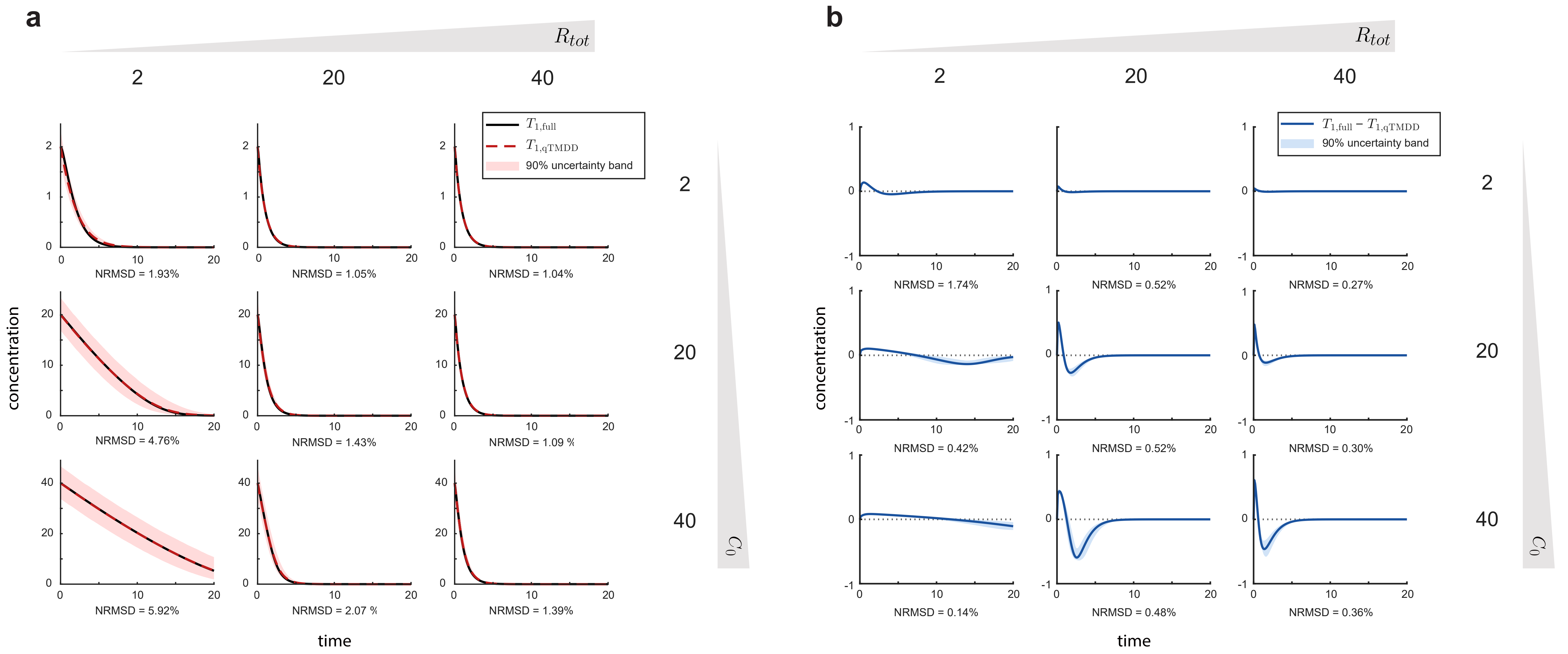

a
b
R_{tot}
2
20
40
C_0
$T_{1,\text{full}}$
$T_{1,\text{qTMDD}}$
90% uncertainty band
$T_{1,\text{full}} - T_{1,\text{qTMDD}}$
concentration
time
NRMSD = 1.93%
NRMSD = 1.05%
NRMSD = 1.04%
NRMSD = 4.76%
NRMSD = 1.43%
NRMSD = 1.09 %
NRMSD = 5.92%
NRMSD = 2.07 %
NRMSD = 1.39%
NRMSD = 1.74%
NRMSD = 0.52%
NRMSD = 0.27%
NRMSD = 0.42%
NRMSD = 0.52%
NRMSD = 0.30%
NRMSD = 0.14%
NRMSD = 0.48%
NRMSD = 0.36%
