# Supplementary material for: Exact Formula of the Total Quasi-Steady State Approximation in Competitive Target-Mediated Drug Disposition
Source: Comput Struct Biotechnol J. 2026 Oct 8;35(1):0223. doi: 10.34133/csbj.0223 (PMC13646460; doi:10.34133/csbj.0223)
Supplement: Supplementary file 1 [file csbj.0223.f1.zip › S5 Table.pdf]

S5 Table. Parallel retries results of NONMEM estimation.

	full TMDD	mTMDD	qTMDD
**Success rate**	63%	43%	69%
**Standard deviation parameter estimates across successful runs**			
Population parameters			
CL	1.94	0.88	2.04
V_C	0.81	0.67	0.85
k_{int}	0.49	0.15	0.52
k_{a_im}	0.0017	0.0008	0.0017
k_{a_sc}	4.3e-05	4.0e-06	4.5e-05
V_D	0.0008	0.0002	0.0001
Inter-individual variability			
CL	0.04	0.03	0.04
VC	0.27	0.25	0.28
Residual error			
proportional error	0.0040	0.0010	0.0042

100 NONMEM estimation runs were performed, with initial parameters randomly sampled from a uniform distribution within ±90% of the final model estimates. Each run was performed with MAXEVAL=9999, allowing up to 9,999 objective function evaluations. We defined a run as successful only if both of the following conditions were met: (i) NONMEM reported successful minimization (minimization_successful = 1), and (ii) the final estimate was not close to a parameter boundary (estimate_near_boundary = 0). For the full TMDD, mTMDD, and qTMDD models, the success rates and the standard deviation of parameter estimates across successful runs were reported.
