# Supplementary material for: Exact Formula of the Total Quasi-Steady State Approximation in Competitive Target-Mediated Drug Disposition
Source: Comput Struct Biotechnol J. 2026 Oct 8;35(1):0223. doi: 10.34133/csbj.0223 (PMC13646460; doi:10.34133/csbj.0223)
Supplement: Supplementary file 1 [file csbj.0223.f1.zip › S6 Table.docx]

| model | mean \|Δ\|r\|\| | median \|Δ\|r\|\| | pairs with increase in \|r\|≥0.1 | pairs with increase in \|r\|≥0.2 |
| --- | --- | --- | --- | --- |
| mTMDD | 0.1483 | 0.1467 | 16 | 8 |
| qTMDD | 0.1013 | 0.0721 | 5 | 3 |

**S6 Table. Differences between correlation matrices of the approximation models and the full TMDD model.** Δ|r| represents the signed change in absolute correlation for each parameter pair, calculated as $\Delta\left| r \right|=\left| r_{approx} \right|-|r_{full}|$.
