# Supplementary material for: Exact Formula of the Total Quasi-Steady State Approximation in Competitive Target-Mediated Drug Disposition
Source: Comput Struct Biotechnol J. 2026 Oct 8;35(1):0223. doi: 10.34133/csbj.0223 (PMC13646460; doi:10.34133/csbj.0223)
Supplement: Supplementary file 1 [file csbj.0223.f1.zip › S9 Fig.pdf]

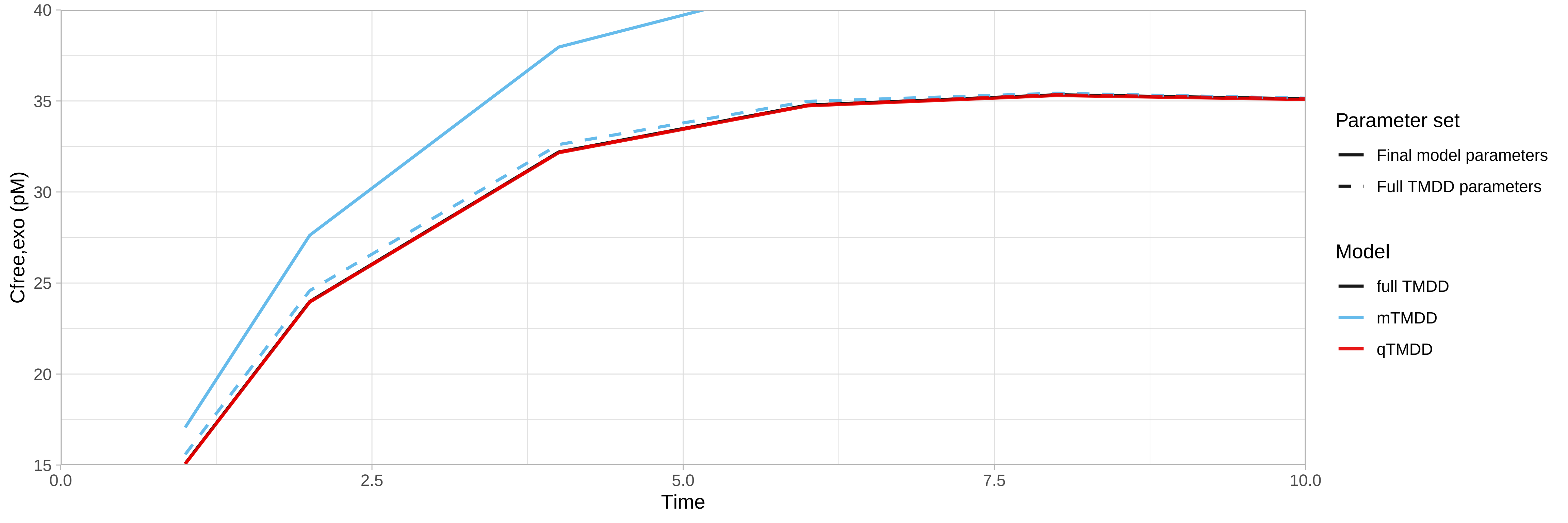
Cfree,exo (pM)
40
35
30
25
20
15
0.0
2.5
5.0
7.5
10.0
Time
Parameter set
Final model parameters
Full TMDD parameters
Model
full TMDD
mTMDD
qTMDD
