# Supplementary material for: Exact Formula of the Total Quasi-Steady State Approximation in Competitive Target-Mediated Drug Disposition
Source: Comput Struct Biotechnol J. 2026 Oct 8;35(1):0223. doi: 10.34133/csbj.0223 (PMC13646460; doi:10.34133/csbj.0223)
Supplement: Supplementary file 1 [file csbj.0223.f1.zip › Supplementary Materials.docx]

Supplementary Materials

**S1 Text. Supplementary materials for competitive qTMDD**

**S2 Text. The NONMEM code of the population pharmacokinetic model with each approximation method for competitive TMDD implemented**

**S1 Fig. Geometry of three real roots of** $\boldsymbol{\Psi}_{\mathbf{1}}\left( \mathbf{x} \right)$ **on the complex plane.** Three real roots $z_{1},z_{2},z_{3}$ of $\Psi_{1}\left( x \right)$ correspond to the real-axis projections of $P$ (blue), $e^{2\pi i/3}P$ (green), $e^{4\pi i/3}P$ (orange), given as $\tilde{z_{j}}/2$ for $j=1,2,3$, respectively; specifically, $z_{j}=-\frac{1}{3a_{1}}\left( b_{1}+\tilde{z_{j}} \right),a_{1}>0$. Thus, a larger real-axis projection implies a smaller root. Since the three complex numbers are separated by 120°, exactly one of them necessarily falls within 60° of the positive horizontal axis (shaded wedge). This number ($P$) has the largest real-axis projection; therefore, it corresponds to the smallest root ($z_{1}$) among the three, and thus the unique biologically meaningful solution.

**S2 Fig. Model structure for cases 1 and 2** k_a_: absorption rate constant of drug from injection site, K_SS2_: equilibrium constant of drug and receptor binding, K_SS2_endo_: ~of endogenous compound and receptor binding, K_SS1_: ~of drug and FcRn binding, CL: apparent clearance of drug at central compartment, V_c_: apparent volume of distribution of drug and endogenous compound at central compartment, k_int_: elimination rate of drug-receptor complex, k_syn_: synthesis rate of endogenous compound, k_el_endo_: elimination rate of unbound endogenous compound, k_int_endo_: elimination rate of endogenous compound-receptor complex, V_D_: apparent volume of distribution of drug at FcRn-mediated recycling site, k_uptake_: uptake rate constant of drug from central to FcRn-mediated recycling site, k_recycle_: recycling rate constant of drug from FcRn-mediated recycling site to central due to its binding with FcRn, receptor: therapeutic receptor of anakinra or rhIL-7-hyFc, FcRn: neonatal Fc receptor.

**S3 Fig. Competitive qTMDD provides an accurate approximation of full competitive TMDD across the tested total receptor concentrations and initial drug concentrations.** (a) Trajectory of $T_{1}=C_{1}+RC_{1}$ simulated from full competitive TMDD (black) and competitive qTMDD (red), across different initial drug ($C_{0}=C_{1,0}=C_{2,0})$ concentration, varied over 2, 20, 40, and total receptor concentration ($R_{tot})$, varied over 2, 20, 40. The other parameters were fixed as $k_{on,1}=1, k_{off,1}=0.5, k_{int,1}=1.1, k_{el,1}=0.01, k_{on,2}=0.3, k_{off,2}=0.4, k_{int,2}=2, k_{el,2}=0.01$. (b) Same as in (a), but trajectory of $C_{1}$ simulated from full competitive TMDD (black) and competitive mTMDD (blue). For all simulated cases, competitive qTMDD approximates full competitive TMDD more accurately than competitive mTMDD.

**S4 Fig. Competitive qTMDD provides a more accurate approximation of full competitive TMDD across the tested initial drug concentrations.** (a) Trajectory of $T_{1}$ simulated from full competitive TMDD (black), competitive qTMDD (red), competitive mTMDD (blue), across different initial exogenous drug ($C_{1,0}$) concentration, varied over 1, 10, 100, and endogenous ligand ($C_{2,0}$) concentration, varied over 1, 10, 100. The other parameters were fixed as parameters used in S3 Fig. (b) Same as in (a), but for trajectories of $T_{2}$. For both $T_{1}$and$T_{2}$, competitive qTMDD approximates full competitive TMDD more accurately than competitive mTMDD.

**S5 Fig. Uncertainty propagation and approximation accuracy of competitive qTMDD across different total receptor and initial drug concentrations.** (a) Black solid line and red dashed line represent the simulated total ligand concentration ($T_{1}=C_{1}+RC_{1}$) over time by the full competitive TMDD and competitive qTMDD models, respectively. Each column represents total receptor concentration $R_{tot}=2,20,40$ and each row represents initial drug concentration $C_{0}=C_{1,0}=C_{2,0}=2,20,40$. The other parameters were fixed as the parameters used in S3 Fig. Red shaded regions represent pointwise 90% uncertainty bands, defined by the 5th and 95th percentiles of 1000 competitive qTMDD simulations. In each simulation, $R_{tot},C_{1,0},C_{2,0}$ were independently perturbed using Gaussian distributions centered at their nominal values, with standard deviation equal to 10% of the corresponding nominal values while all other parameters were fixed. For each simulation, the root mean square deviation from the nominal qTMDD trajectory was normalized by the maximum $T_{1}$ concentration of the nominal qTMDD trajectory. The median of the resulting 1000 normalized root mean square deviations (NRMSD) is indicated below each graph. (b) Same as in (a), but showing the paired concentration difference, $T_{1,full}-T_{1,qTMDD}$. Blue solid lines and light blue shaded regions represent the nominal differences and their pointwise 90% uncertainty bands, respectively. The NRMSD indicated below each graph was normalized by the maximum $T_{1}$ concentration of the simulated full TMDD trajectory. The maximum median paired NRMSD was 1.74%, demonstrating that competitive qTMDD maintained its approximation accuracy for all tested cases despite uncertainty in the initial ligand and receptor concentrations.

**S6 Fig. The exact formula of competitive qTMDD provides a faster and more stable computation than using the Newton-Raphson method.** Violin plots extend Fig. 3b by additionally including the Newton–Raphson method initialized at $T_{1}/2$. The exact formula achieved shorter computation times than the Newton–Raphson method. In addition, the Newton–Raphson method showed unstable root-finding performance, with convergence (green) and non-convergence (red) alternating depending on the parameter conditions.

**S7 Fig.** **Goodness-of-fit plots of (a) Case 1 and (b) Case 2.** (A, E) population prediction vs observations, (B, F) individual predictions vs observations, (C, G) time vs conditional weighted residuals, (D, H) population prediction vs conditional weighted residuals. The fitted lines of the observation vs. prediction closely matched the y=x line (A, B, E, F), and most conditional weighted residuals (CWRES) were within the range of −2 to 2 with the fitted line positioned close to zero (C, D, G, H).

**S8 Fig.** **Visual predictive checks of (a) Case 1 and (b) Case 2.** Observed 5^th^, 50^th^, 95^th^ percentiles are compared with 90% prediction intervals from 200 simulations. No prediction correction was applied. This VPC plot showed that the prediction intervals adequately encompassed all observations without excessive overlap between intervals, indicating that the model appropriately captured both the central tendency and variability of the data.

**S9 Fig.** **Enlarged early-phase drug concentration profiles for Case 2 simulated using the estimated parameters.** Free drug concentration profiles were simulated at the population level using the full TMDD (black), qTMDD (red), and mTMDD (blue) models. Solid lines indicate simulations using parameter estimates obtained from the corresponding model, whereas dotted lines indicate simulations using the parameter estimates obtained from the full TMDD model.

**S10 Fig.** **Comparison of parameter correlation matrices for the full TMDD, qTMDD, and mTMDD models**. Pairwise correlation coefficients were extracted from the NONMEM .cor files. The coefficients obtained from the full TMDD model were plotted against those from the mTMDD (left) and qTMDD (right) models to assess agreement in the parameter correlation structures. Nine parameters were freely estimated, resulting in 36 unique off-diagonal parameter pairs for each model.

**S11 Fig. Differences in profile likelihoods between the approximated and full TMDD models.** (a) Two-dimensional ΔOFV surfaces across k_on_ and k_off_ for qTMDD (left) and mTMDD (right). (b) ΔOFV profiles of K_ss_endo_ and (c) R_tot_ for mTMDD (blue) and qTMDD (red). ΔOFV was calculated at each parameter setting as OFV_approximation model_ – OFV_full TMDD_

**S1 Table. Parameter estimate results, OFV values and elapsed time** CL: Apparent clearance of drug at central compartment, V_c_: Apparent volume of distribution of drug and endogenous compound at central compartment, k_a_: Absorption rate constant of drug from injection site (im for intramuscular injection, sc for subcutaneous injection), k_int_: elimination rate of drug-receptor complex, k_syn_: synthesis rate of endogenous compound and V_D_: Apparent volume of distribution of drug at FcRn-mediated recycling site. Values are point estimates; numbers in parentheses are the relative standard error for estimation results (RSE, %) and standard deviations for elapsed time (seconds).

**S2 Table. Parameter estimate results and OFV values for Case 2 with dense simulated dataset** Values are point estimates; numbers in parentheses are the relative standard error (RSE, %). Values are point estimates; numbers in parentheses are the relative standard error (RSE, %). Refer to Table S1 for parameter definitions and details.

**S3 Table. Dataset for Case 1**

**S4 Table. Dataset for Case 2**

**S5 Table. Parallel retries results of NONMEM estimation.** 100 NONMEM estimation runs were performed, with initial parameters randomly sampled from a uniform distribution within ±90% of the final model estimates. Each run was performed with MAXEVAL=9999, allowing up to 9,999 objective function evaluations. We defined a run as successful only if both of the following conditions were met: (i) NONMEM reported successful minimization (minimization_successful = 1), and (ii) the final estimate was not close to a parameter boundary (estimate_near_boundary = 0). For the full TMDD, mTMDD, and qTMDD models, the success rates and the standard deviation of parameter estimates across successful runs were reported.

**S6 Table. Differences between absolute correlation matrices of the approximation models and the full TMDD model.** Δ|r| represents the signed change in absolute correlation for each parameter pair, calculated as $\Delta\left| r \right|=\left| r_{approx} \right|-|r_{full}|$.

**S1 File. qTMDD.zip**
